# Grapevine genome analysis demonstrates the role of gene copy number variation in the formation of monoterpenes

**DOI:** 10.3389/fpls.2023.1112214

**Published:** 2023-03-16

**Authors:** Robin Nicole Bosman, Jessica Anne-Marie Vervalle, Danielle Lisa November, Phyllis Burger, Justin Graham Lashbrooke

**Affiliations:** ^1^ South African Grape and Wine Research Institute, Stellenbosch University, Stellenbosch, South Africa; ^2^ Laboratory of Nematology, Department of Plant Sciences, Wageningen University, Wageningen, Netherlands; ^3^ Department for Crop Development, Agricultural Research Council - Infruitec-Nietvoorbij, Stellenbosch, South Africa

**Keywords:** terpenes, TPS, grapevine, gene copy number, genomics, QTL, VOC

## Abstract

Volatile organic compounds such as terpenes influence the quality parameters of grapevine through their contribution to the flavour and aroma profile of berries. Biosynthesis of volatile organic compounds in grapevine is relatively complex and controlled by multiple genes, the majority of which are unknown or uncharacterised. To identify the genomic regions that associate with modulation of these compounds in grapevine berries, volatile metabolic data generated *via* GC-MS from a grapevine mapping population was used to identify quantitative trait loci (QTLs). Several significant QTLs were associated with terpenes, and candidate genes were proposed for sesquiterpene and monoterpene biosynthesis. For monoterpenes, loci on chromosomes 12 and 13 were shown to be associated with geraniol and cyclic monoterpene accumulation, respectively. The locus on chromosome 12 was shown to contain a geraniol synthase gene (*VvGer*), while the locus on chromosome 13 contained an α-terpineol synthase gene (*VvTer*). Molecular and genomic investigation of *VvGer* and *VvTer* revealed that these genes were found in tandemly duplicated clusters, displaying high levels of hemizygosity. Gene copy number analysis further showed that not only did *VvTer* and *VvGer* copy numbers vary within the mapping population, but also across recently sequenced *Vitis* cultivars. Significantly, *VvTer* copy number correlated with both *VvTer* gene expression and cyclic monoterpene accumulation in the mapping population. A hypothesis for a hyper-functional *VvTer* allele linked to increased gene copy number in the mapping population is presented and can potentially lead to selection of cultivars with modulated terpene profiles. The study highlights the impact of *VvTPS* gene duplication and copy number variation on terpene accumulation in grapevine.

## Introduction

Terpenes are one of the largest classes of metabolites in plants, where they serve various primary and specialized roles. Volatile terpenes, such as monoterpenes and sesquiterpenes, mainly function as specialized metabolites and are involved in plant-pathogen interactions, protection of plants against herbivores, and are also produced to attract pollinators and seed-dispersing animals (Dudareva et al., 2013; Vranová et al., 2013). Together with additional volatile organic compounds (VOCs) such as short-carbon chain compounds (green leaf volatiles), C13-norisoprenoids and methoxypyrazines, they contribute to the varietal aroma of grape berries (Dunlevy et al., 2009). Indeed, monoterpenes and sesquiterpenes have been extensively studies for their contribution to the distinctive varietal aroma of aromatic cultivars such as Muscat-cultivars (monoterpene alcohols including linalool, geraniol, and α-terpineol), ‘Shiraz’ (the sesquiterpene rotundone), ‘Riesling’ and ‘Gewürztraminer’ (the monoterpene rose-oxide) (Dunlevy et al., 2009).

Biosynthesis of monoterpenes and sesquiterpene occurs *via* the methyl-erythritol-phosphate (MEP) and mevalonic acid (MVA) pathways, respectively. The first step in the MEP pathway is catalysed by 1-deoxy-D-xylylose-5-phosphate synthase (DXS), an enzyme which is considered to be the vital rate-limiting enzyme in plastidial terpene biosynthesis(Tholl, 2015; Bosman and Lashbrooke, 2023). Several QTL mapping and association studies in grapevine have identified a singular SNP in the active site of *VvDXS1* as a causal mutation for increased monoterpene content in Muscat cultivars (Doligez et al., 2006; Battilana et al., 2009; Emanuelli et al., 2010). A SNP at position 1822 of *VvDXS1* (G>T) in Muscat cultivars causes a non-synonymous substitution of a lysine (K) with an asparagine (N) at position 284 of the VvDXS1 protein. Functional characterisation of VvDXS1 showed that the non-synonymous amino acid substitution influences enzyme kinetics by increasing the catalytic efficiency of VvDXS1, thereby increasing the total monoterpene content of cultivars carrying this SNP (Battilana et al., 2011).

While VvDXS1 is able to regulate total monoterpene accumulation *via* biosynthesis of the prenyldiphosphate precursors, terpene synthases (TPSs) are responsible for the formation of specific terpenes (Steele et al., 1998; Chen et al., 2011). Monoterpene synthases catalyse the coupled ionisation, isomerisation and cyclisation of geranyldiphosphate (GPP) leading to the formation of a reactive carbocation intermediate and subsequent reactions e.g. deprotonation or ring closures will form the final monoterpene product (Davis and Croteau, 2000; Degenhardt et al., 2009).

The *Vitis vinifera* reference genome, PN40024, has a greatly expanded TPS gene family, with an initial prediction of 152 loci and 69 putatively functional TPSs (Jaillon et al., 2007; Martin et al., 2010). However, the recent availability of phased diploid grapevine genomes of various cultivars (Minio et al., 2019; Zhou et al., 2019; Massonnet et al., 2020) reveals that the TPS gene family size varies greatly between cultivars (Smit et al., 2020). Furthermore, *VvTPS*s are organised in large tandemly duplicated clusters, and a great portion of genes are hemizygous (Martin et al., 2010; Jiang et al., 2019; Smit et al., 2020). Recent research has also shown that grapevine has cultivar-specific *TPS* genes (Drew et al., 2016; Smit et al., 2019). Cultivar-specific *TPS*s arise due to small sequence variations, such as single nucleotide polymorphisms (SNPs), which cause a functional change of the enzyme. The extensive level of duplication and functional plasticity of VvTPSs contribute to the neofunctionalisation of these enzymes and results in the large diversity in metabolites formed by VvTPSs (Bosman and Lashbrooke, 2023).

This study utilises a biparental grapevine cross population established by crossing a wine cultivar and a table grape cultivar. A dense linkage map has previously been created for this mapping population (Vervalle et al., 2022) which segregates for various traits, including aromatic profile. Quantification of volatile organic compounds in this population over several seasons was performed and genomic regions associated with these compounds identified. Genomic regions containing multiple *TPS* genes were further interrogated, and the large variety in cultivar specific *TPS* copy number associated with accumulation of specific monoterpenes characterised.

## Materials and methods

### Plant materials and sampling

82 progenies of the mapping population (‘Deckrot’ x G1-7720), which is held at the Agricultural Research Council (ARC) Nietvoorbij (Stellenbosch, South Africa, 33° 54’ 47.6’’ S, 18° 51’ 54.9’’ E) were used for analysis. G1-7720 is a table grape selection developed by the ARC and is a cross between ‘Black Rose’ and ‘Muscat Seedless’. Grape berries from the progenies and parents were sampled at veraison (EL-stage 35) in January 2019, and at harvest ripeness (EL-stage 38) in February 2021 and 2022. Veraison berries from each bunch were further divided into “pre-veraison” (berries that were still green and firm) and “veraison” (berries which had changed colour and softened). The skin and flesh of all berries (pre-veraison, veraison and harvest ripe) were separated. Additionally, the parent cultivars were sampled at various early developmental stages (EL-stages 19, 23, 26, 29, 31 and 33) between October-December 2020. Three biological replicates were sampled for both cultivars at each developmental stage. Flowers and berries were removed from the rachis, and the rachis was discarded. All plant material was frozen in liquid nitrogen, ground to a fine powder and stored at -80°C.

### Volatile organic compound analysis

Approximately 150 mg of ground frozen berry skin tissue was weighed into a 20-mL GC vial and 2 ml tartaric acid buffer (5 g.L^-1^ tartaric acid, 2 g.L^-1^, 0.8 g.L^-1^ and 4.28 M NaCl; pH 3.2), containing 5 g.L^-1^ internal standard (3-octanol), was added to the vial. Headspace solid phase microextraction (HS-SPME) and gas chromatography mass spectrometry (GCMS) were performed according to the method described in Joubert et al. (2016). Samples were extracted from the vial head space using a 50/30 µm grey divinylbenzene/carboxen/polydimethylsiloxane (DVB/CAR/PDMS) fiber (Supelco, USA). The GCMS analysis was carried out on an Agilent 7890B GC equipped with a 5977B MSD and a PAL RSI 85 autosampler. Chromatographic separation was achieved using a HP-5MSUI capillary column (30m x 0.25 µm x 0.25 mm). The purge flow was 30 mL.min^-1^ (for 90 seconds). The oven parameters were as follows: initial temperature of 40°C (2 min), a linear increase to a final temperature of 250°C (at a rate of 6°C.min^-1^), and the temperature was held at 250°C for a final 5 min. The MS detector was operated in scan mode (from 35 to 350 m/z).

Agilent MassHunter Qualitative and Quantitative software packages were used for data analysis. Volatile compounds were identified according to their elution times and masses compared to those of respective authentic standards. Compounds without available authentic standards were identified by matching their mass spectrum with the NIST (Linstrom and Mallard, 2001) mass spectral library, in combination with Kovatz retention indices (RIs). Relative quantification of the compounds was achieved by normalising the peak area of each compound with the peak area of the internal standard. Concentrations are expressed as µg of 3-octanol equivalents per gram fresh weight.

For α-terpineol and geraniol, quantification was achieved through external standard calibration which was done by plotting standard curves using the internal response ratio versus the standard concentration. The resultant concentrations in μg.L^-1^ were then normalised to the berry fresh weight to obtain the concentration (in µg.g^-1^ FW).

### QTL mapping

A total of 137 progeny of the ‘Deckrot’ x G1-7720 mapping population have previously been genotyped with the Vitis18K SNP chip and 92 simple sequence repeat (SSR) markers (Vervalle et al., 2022). The genotyping data were used to construct parental linkage maps for ‘Deckrot’ and G1-7720 with JoinMap^®^5 (Van Ooijen, 2006). Both maps represented all 19 linkage groups of grapevine and contained 1910 and 2252 markers in the maternal and paternal maps respectively. The maps displayed an average inter-locus gap distance of 0.80 cM. The genetic maps were combined with the metabolomic data to perform QTL analyses in MapQTL^®^6 (Van Ooijen, 2009). QTL regions were first identified through interval mapping with the maximum likelihood mixture model algorithm. Subsequently, regions were further defined with the multiple-QTL models (MQM) mapping. Genome-wide significant LOD thresholds were determined with permutation tests of 1000 permutations each. All maps were drawn with MapChart v2.32 (Voorrips, 2002).

### Genomic analysis of significant QTLs

The position of significant QTLs on the grapevine reference genome (PN40024) version 2 (Jaillon et al., 2007) was determined from the physical positions of the two neighbouring flanking markers. Annotated genes within each QTL were retrieved from URGI (https://urgi.versailles.inra.fr/Species/Vitis/Annotations) and is based on the VCost v.3 structural annotation of the 12X.2 reference genome (Jaillon et al., 2007; Canaguier et al., 2017). Gene function was predicted *via* BLASTp analysis (https://blast.ncbi.nlm.nih.gov/Blast.cgi) by querying protein sequences against the UniProtKB/SwissProt database and selecting the top hit for each sequence. Candidate genes were selected based on evidence from literature and their function was further investigated through molecular phylogeny. Phylogenetic trees were created on “Geneious Tree Builder” 2022.0.1 (https://www.geneious.com) using the default ‘Geneious Tree Builder” function which used the UPGMA method with the Jukes-Cantor distance measure algorithm and 100 bootstrapping replicates. Multiple sequence alignments were created using the default ‘Geneoius Alignment’ setting. Candidate gene expression was compared to metabolite data using the Transcriptomics & Metabolomics integrated database (TransMetaDb) (Savoi et al., 2016; Savoi et al., 2017) available on the Vitis Visualization platform (VitViz) (http://www.vitviz.tomsbiolab.com/).

### TaqMan SNP genotyping assay

A custom TaqMan SNP genotyping assay (ThermoFisher Scientific) was designed for the *VvDXS1* SNP and used to genotype the mapping population under investigation. Genomic DNA was extracted from grape berry skins as described in Reid et al. (2006) however the DNase treatment step was substituted with a RNase treatment to eliminate RNA. The custom assay mix consisted of 1X TaqMan^®^ MasterMix (ThermoFisher Scientific), 1X Custom Assay mix which contains the custom primers and probes (**Table S1**), and 1 µL (10-40 ng) of genomic DNA. Primers and probes were designed using PrimerExpress (Singh and Pandey, 2015). The assay was performed in QuantStudio 3 Real-Time PCR System with the following conditions: a pre-read stage for 30 seconds at 60°C, initial denaturation at 95°C for 5 minutes, 40 cycles of denaturation (95°C for 15 seconds) and annealing/extension (60°C for 1 minute), and then a post-read stage for 30 seconds at 60°C.

Confirmation of the results from the TaqMan assay was performed for a subset (20 progeny and the parents) of the mapping population. *VvDXS1* was isolated from gDNA *via* polymerase chain reaction (PCR). The reaction mixture (25 μL) contained 0.2 μM of the forward (5’-GTCATAGGTGATGGAGCCA-3’) and reverse (5’-ATCTTACCTGTTCTGTCTAGC-3’) primers (Emanuelli et al., 2014), 1 μL DNA (approximately 50 ng) and 1X GoTaq^®^Green Master Mix (Promega, USA). PCR products were visualised on a 1% agarose gel, excised and purified using Zymoclean Gel DNA recovery kit, and subsequently sequenced *via* Sanger sequencing.

### Analysis of gene copy number variation

Total genomic DNA was extracted from pre-veraison samples as described in in Reid et al. (2006), and relative gene copy number was determined *via* a qPCR-based method described previously (Ma and Chung, 2014; Bhattacharya et al., 2019). qPCR was performed in a QuantStudio 3 Real-Time PCR System (ThermoFisher) in a 15 µL reaction mixture which contained 1X SYBR (Power SYBR Green, Applied Biosystems), 0.2 µM of each primer pair, 2 µL of genomic DNA (approximately 90 ng) and nuclease-free water. Reactions were repeated in technical triplicate for each gene-sample combination, making use of *VvActin* primers to normalise for the amount of genomic DNA assayed in each sample and either *VvGer* or *VvTer* gene specific primers for amplification of the respective gene copies (for primer sequences see **Table S2**). The PCR conditions were: initial denaturation step at 95°C for 3 minutes, 40 cycles (95°C for 3 seconds and 60°C for 20 seconds), and melt curve analysis from 60 to 95°C. Relative gene copy number was determined with the 2^−ΔΔCt^ method (Ma and Chung, 2014), making use of the QuantStudio Design and Analysis Desktop Software package (v1.5.1) from Applied Biosystems for processing relative copy number and 95% confidence intervals. All gene copy numbers are reported relative to the gene copies present in the ‘Deckrot’ cultivar.

Correlation analysis, Pearson’s correlation (R) and Spearman’s rank correlation (ρ), between expression, copy number and metabolite data was performed with XLStat (version 20213.1) add-on for Excel (Addinsoft, 2023).

### Gene expression quantification

Total RNA was extracted from pre-veraison samples using the Spectrum™ Plant Total RNA Kit (Sigma-Aldrich) with the removal of genomic DNA *via* on-column DNase digestion using the On-Column DNase I Digestion Set (Sigma-Aldrich) according to the manufacturer’s instructions. 1 µg of RNA was converted to cDNA using the GoScript™ Reverse Transcription mix, Oligo(dT) (Promega).

The relative expression of genes was measured *via* qPCR using primers from literature or newly designed primers (**Table S2**) with *VvActin* used as an endogenous control. qPCR was performed as described for gene copy number variation analysis above. Gene expression data for the progeny is reported relative to the individual with the lowest expression, while gene expression data for the parents’ developmental stages are reported relative to gene expression in ‘Deckrot’ green berry stage (EL-stage 33).

### Gene isolation, transformation, sequencing

Genes were isolated from cDNA samples *via* polymerase chain reaction (PCR) using primers listed in **Table S2**. The PCR mixture (25 μL) contained 0.2 μM of the forward primer and reverse primers, 5 μL cDNA (1:10 dilution), 1X ExTaq Buffer and 1U ExTaq polymerase (Takara). The reaction was performed in an Applied Biosystems™ MiniAmp thermal cycler (ThermoFisher Scientific) and conditions were as follows: initial denaturation at 95°C for 3 minutes, then 40 cycles (95°C for 30 seconds, 55°C for 30 seconds, 72°C for 2 minutes) and a final elongation step at 72°C for 7 minutes. PCR products were purified using Zymoclean Gel DNA Recovery Kit (Zymo Research) as per the manufacturer’s protocol.

PCR products were ligated into pGEM^®^ T-Easy Vector System (Promega) overnight at 4°C. Ligated vectors were subsequently transformed into competent DH5α *E. coli* cells *via* heat-shock. Transformed cells were plated on LB agar containing ampicillin and after overnight incubation at 37°C, colonies were screened *via* PCR using M13 primers. Plasmids were isolated from positive colonies using GenElute™ Plasmid Miniprep Kit (Sigma-Aldrich), as per the manufacturer’s protocol. Sanger sequencing was performed by the Central Analytical Facilities at Stellenbosch University (Stellenbosch, South Africa).

## Results

### The parents of the cross population differ for terpene accumulation

**Figure 1:**
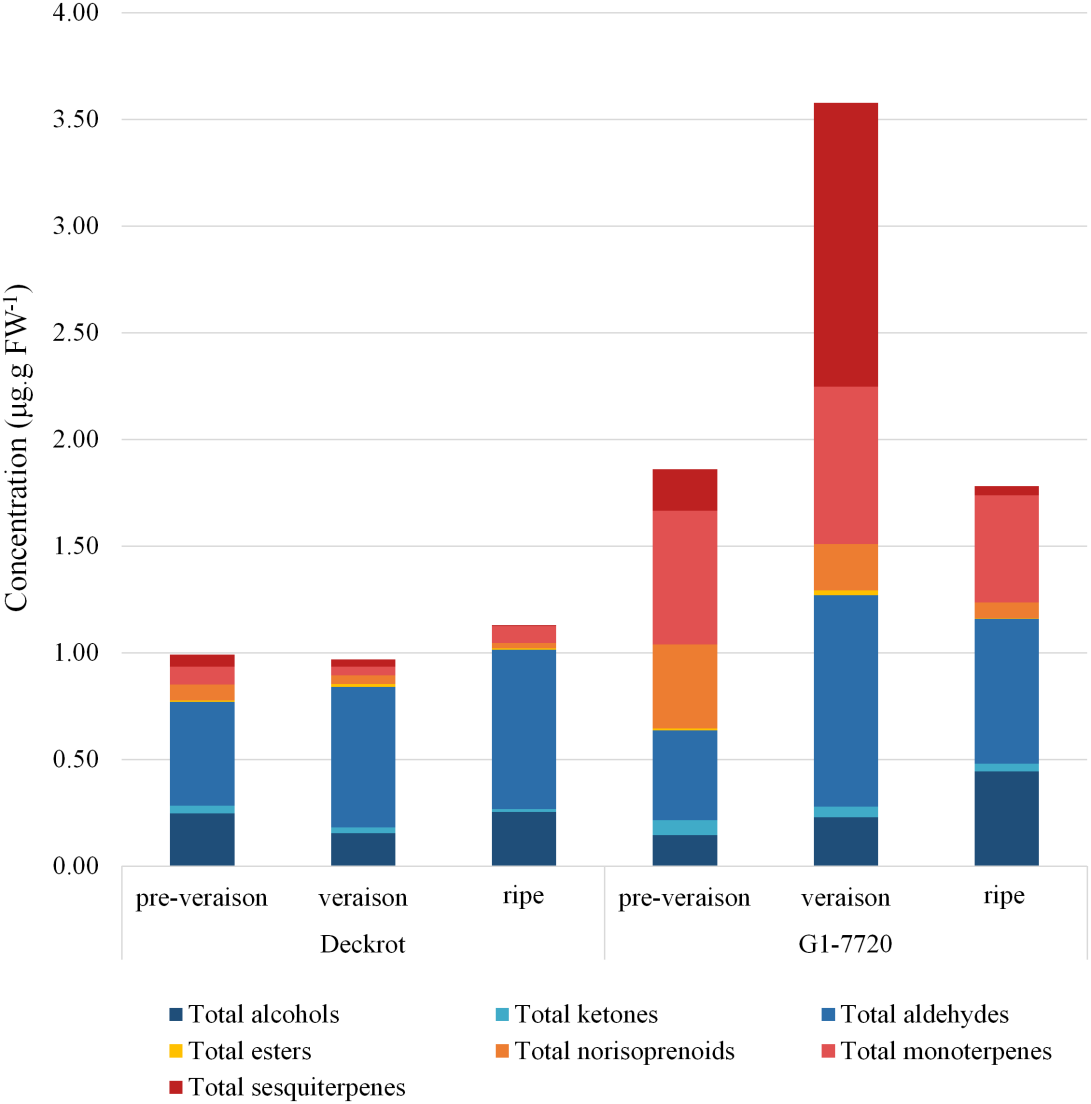
The volatile composition of the parent cultivars ‘Deckrot’ and G1-7720 during pre-veraison, veraison and harvest ripe stage.

68 different volatile compounds were identified from the GCMS analysis: six alcohols, three ketones, ten aldehydes, two esters, four C13-norisoprenoids, 18 monoterpenes and 25 sesquiterpenes (**Table S3**). The volatile profiles of the parent cultivars were compared (**Figure 1**) and show clear segregation. Overall, the table grape selection, G1-7720, which is categorised as having a muscat aroma (Vervalle et al., 2022), has a higher volatile content regardless of developmental stage. While, the terpene content of both parents is highest at veraison, G1-7720 has a significant increase in sesquiterpene content from pre-veraison to veraison berries. G1-7720 shows significantly higher terpene levels than that of ‘Deckrot’. Aldehydes and alcohols also make up a major proportion of the volatile composition of each cultivar, specifically ‘Deckrot’, with hexanal, *trans*-2-hexenal and (*E*)-3-hexen-1-ol being the most significant contributors.

The distribution of the various volatile compound classes in the progeny is shown in **Figure S1**. Aldehydes are the most abundant compound class in the population; the average ratio of total aldehydes to total VOCs ranges between 34-47% across the various developmental stages. *Trans*-2-hexenal (an aldehyde) is the most abundant compound in the population. In fact, all six-carbon volatiles, also known as green leaf volatiles (GLVs), are among the most abundant VOCs within the population in all development stages. Sesquiterpenes are the most diverse class of compounds identified (26 compounds) in this study however they are present in low concentrations during most years, with the highest concentrations found in unripe veraison berries.

### Candidate genes for were identified in several genomic regions associated with volatile organic compounds

**Figure 2:**
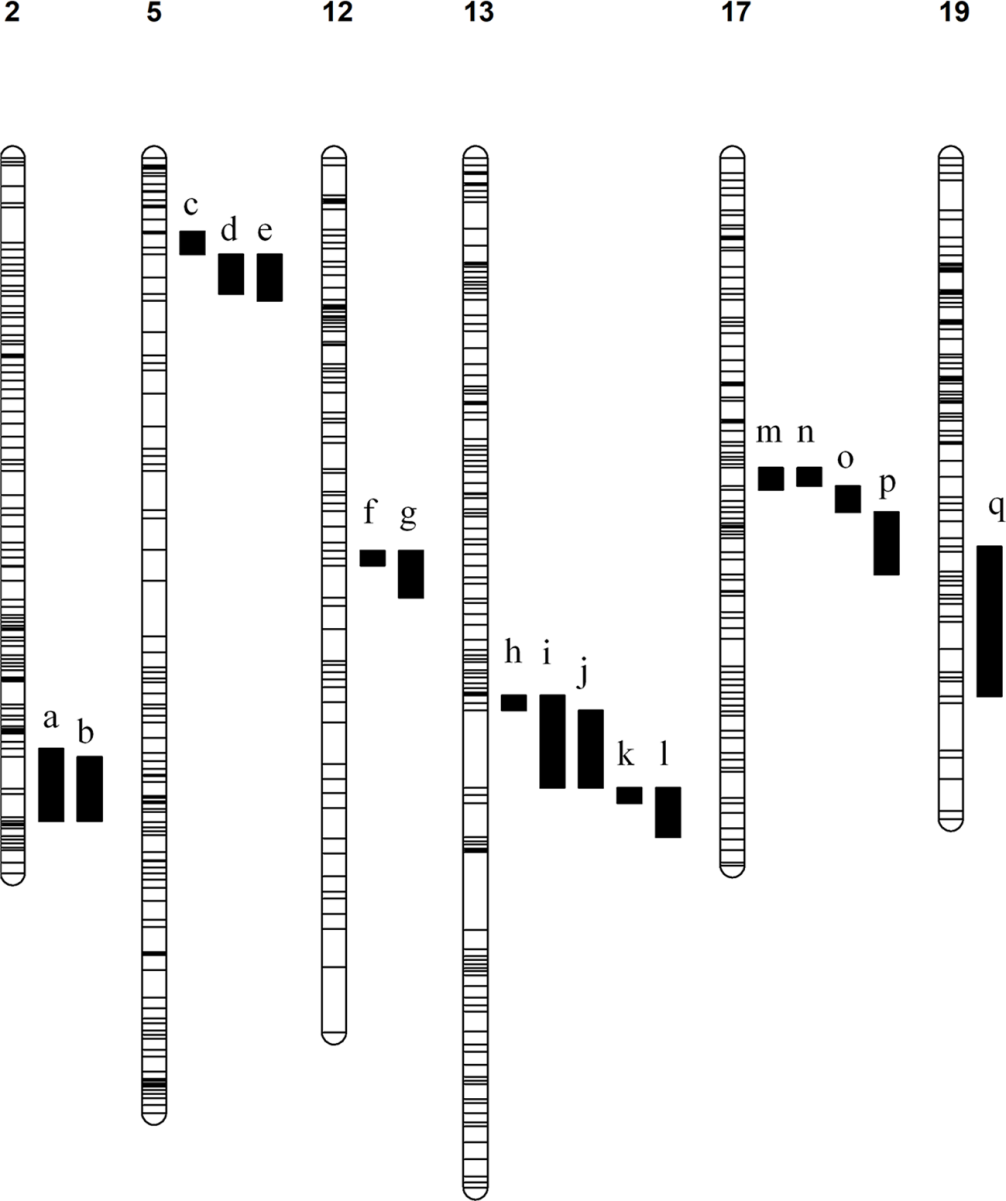
Linkage maps with associated QTLs. The chromosome number is indicated above each map and all QTLs are numbered a – q. The associated compounds for each QTL are listed. a) aldehydes total (veraison 2019) and trans-2-hexenal (veraison 2019). b) aldehydes total (2022) and trans-2-hexenal (2022). c) E-citral (veraison & 2021), β-citronellol (pre-veraison & 2022), cis-rose oxide (veraison, 2021 & 2022), geraniol (2022), limonene (2022), linalool (2022), total monoterpenes (2022), nerol oxide (veraison), p-mentha-1,5-dien-8-ol (veraison & 2021), trans-β-Ocimene (2022), α-terpinene (2022), α-terpineol (2022), α-terpinolene (2022), β-Myrcene (2022), γ-Terpinene (2022), p-Cymene (2022). d) 6-Methyl-5-hepten-2-one (pre-veraison, veraison, 2021), cis-rose oxide (pre-veraison), linalool (pre-veraison), p-Cymene (pre-veraison & veraison), p-mentha-1,5-dien-8-ol (pre-veraison), trans-β-Ocimene (pre-veraison & veraison), α-terpinene (veraison), γ-Terpinene (pre-veraison & veraison). e) β-Citronellol/Nerol (pre-veraison & 2021), C13-norisoprenoids total (veraison & 2021), geraniol (pre-veraison, veraison & 2021), limonene (pre-veraison, veraison & 2021), linalool (2021), linalool oxide (pre-veraison, veraison & 2021), monoterpenes total (pre-veraison, veraison & 2021), nerol oxide (pre-veraison & veraison), p-Cymene (2021), Terpinene-4-ol (pre-veraison, veraison & 2021), trans-β-Ocimene (2021), α-terpinene (veraison & 2021), α-Terpineol (pre-veraison, veraison & 2021), α-terpinolene (pre-veraison, veraison & 2021), β-Myrcene (pre-veraison, veraison & 2021), γ-Terpinene (2021). f) Geraniol (pre-veraison) & β-Myrcene (pre-veraison). g) Geraniol (veraison) & β-Myrcene (veraison). h) α-Terpineol (veraison) & α-terpinolene (veraison). **(I)** Terpinene-4-ol (2021). j) 1,8-Cineole (pre-veraison & veraison), limonene (veraison), nerol oxide (veraison), p-Cymene (veraison & 2021), p-mentha-1,5-dien-8-ol (pre-veraison & veraison), Terpinene-4-ol (pre-veraison & veraison), α-terpinene (pre-veraison & veraison), γ-Terpinene (pre-veraison). k) limonene (pre-veraison), α-terpinene (2021), γ-Terpinene (veraison). l) p-Cymene (pre-veraison), p-mentha-1,5-dien-8-ol (2021), α-terpinolene (pre-veraison). m) cis-rose oxide (2021) & nerol oxide (veraison). n) nerol oxide (pre-veraison). o) cis-rose oxide (veraison). p) E-citral (2021) & cis-rose oxide (pre-veraison). q) α-Cubebene (pre-veraison & veraison) & trans-caryophyllene (pre-veraison and veraison).

186 Significant QTLs were identified associated with 54 compounds across the various seasons under analysis (**Table S4**). Consistent QTLs which were identified in at least two stages were mapped to the PN40024 v2 genome and annotated genes in each region were counted and inspected manually for their potential to regulate the associated metabolic phenotypes. QTLs associated with various monoterpenes were identified on chromosomes 5, 12, 13 and 17, while a QTL associated with the sesquiterpenes trans-caryophyllene and α-cubebene was identified on chromosome 19 and a QTL for total aldehydes and trans-2-hexenal was identified on chromosome 2 (**Figure 2**). Inspection of these genomic regions identified an average of 370 candidate genes in each QTL (**Table 1** **)**.

**Table 1:** List of candidate genes for significant QTLs associated with VOCs.

Linkage group	Compounds	12xV2 position (bp)^a^	Number of genes	Candidate gene	Accession number	Function (based on UniProt/SwissProt hit)
chr02	Total aldehydes Trans-2-hexenal	9086122 - 15054521	335	*VvSAD*	Vitvi02g01527	Stearoyl-[acyl-carrier-protein] 9-desaturase 6 (SAD)
chr05	Several monoterpenes ^b^ Total monoterpenes Geranyl acetone 6-Methyl-5-hepten-2-one Total C13-norisoprenoids	2217503 - 4373666	214	*VvDXS1*	Vitvi05g00372	1-deoxy-D-xylulose-5-phosphate synthase (DXS)
chr12	Geraniol and β-myrcene	7725827-9589001	225	*VvTPS52/VvGer*	Vitvi12g02178	Geraniol terpene synthase
chr13	Several cyclic monoterpenes ^c^	18786002-21772519	201	*VvTPS39/VvTer*	Vitvi13g01307	α-terpineol synthase
chr17	Cis rose-oxide Nerol oxide E-citral	5844409 - 8181258	262	*VvSDR1* *VvSDR2* *VvSDR3* *VvSDR4* *VvSDR5*	Vitvi17g00538 Vitvi17g00537 Vitvi17g00534 Vitvi17g00535 Vitvi17g01453	Short chain dehydrogenase/reductase (SDR)
chr19	*Trans-*Caryophyllene α-Cubebene	8950369-20638362	984	*VvTPS29*	Vitvi19g00956	Germacrene D synthase

a These positions provide a consensus region for overlapping QTLs on the same chromosome. **Table S4** shows the exact position of each compounds’ associated QTL.

b α-terpinene, p-Cymene, trans-β-Ocimene, γ-Terpinene, Linalool Oxide, α-terpinolene, Linalool, cis-Rose oxide, Terpinene-4-ol, α-Terpineol, Geraniol, Limonene, β-Citronellol/Nerol, (E)-Citral, β-myrcene.

c α-terpinene, p-Cymene, 1,8-Cineole, γ-Terpinene, α-terpinolene, Terpinene-4-ol, α-Terpineol.

A QTL on chromosome 2 associated with both total aldehydes and trans-2-hexenal and contained 335 genes. A Stearoyl-[acyl-carrier-protein] 9-desaturase 6 (SAD) was identified as candidate gene from this region. The *Vitis vinifera* SAD (VvSAD) was compared to other functionally characterised SAD enzymes from various species (**Figure S2** and **Table S5**) to infer function.

Three geraniol derived compounds, namely cis-rose oxide, (E)-citral and nerol oxide associated with a QTL on chromosome 17. The enzymes involved in conversion of geraniol to cis-rose oxide, (E)-citral and nerol oxide are not described in grapevine however the reaction likely starts with the reduction of geraniol by an unknown reductase (Lin et al., 2019). To that end five short-chain dehydrogenase/reductases (*VvSDR1-5*) which co-localises with this QTL were selected as candidate genes. Comparison of *VvSDR1-5* expression to metabolite data using TransMetaDb showed that *VvSDR4* and *VvSDR5* had strong positive correlation with citronellol accumulation (R = 0.71 and 0.70, respectively), while *VvSDR1* had a negative correlation (R = -0.54) with citronellol. Additionally, *VvSDR3* had a negative correlation (R = -0.62) with nerol. Molecular phylogenetic analysis of the protein sequences of the identified VvSDRs were compared to that of other plant SDRs (**Figure S3** and **Table S5**), and it was found that the VvSDRs cluster with three nepetalactol synthases which catalyse the conversion of 8-oxogeranial to nepetalactol in catmint (Lichman et al., 2019).

While total sesquiterpenes did not produce any significant QTLs, the sesquiterpenes α-cubebene and *trans*-caryophyllene associated with a QTL on chromosome 19. This QTL co-localises with two predicted sesquiterpene synthases (*VvTPS29* and *VvTPS69*), however *VvTPS69* represented a partial gene and was thus not considered a candidate.

The QTLs associated with monoterpenes predominantly localised to chromosomes 5, 12 and 13. The majority of monoterpenes associated with a QTL region on chromosome 5 which co-localises with *VvDXS1.* Similarly, total monoterpenes and total C_13_-norisoprenoids, localised to the same region on chromosome 5. While the C13-norisoprenoid 6-Methyl-5-hepten-2-one (MHO) also showed a QTL on the same region on chromosome 5.

The acyclic monoterpenes geraniol and β-myrcene associated with an additional QTL on chromosome 12 while several cyclic monoterpenes associated with an additional QTL on chromosome 13. Genomic inspection of these QTLs found that they co-localise with clusters of terpene synthases (*TPSs*). When compared to the PN42004 v2 reference genome the QTL on chromosome 12 was found to co-localise with a cluster of eight TPS genes, while the QTL on chromosome 13 was found to co-localise with a TPS cluster that contains 11 terpene synthases (summarised in **Table S6**).

**Figure 3:**
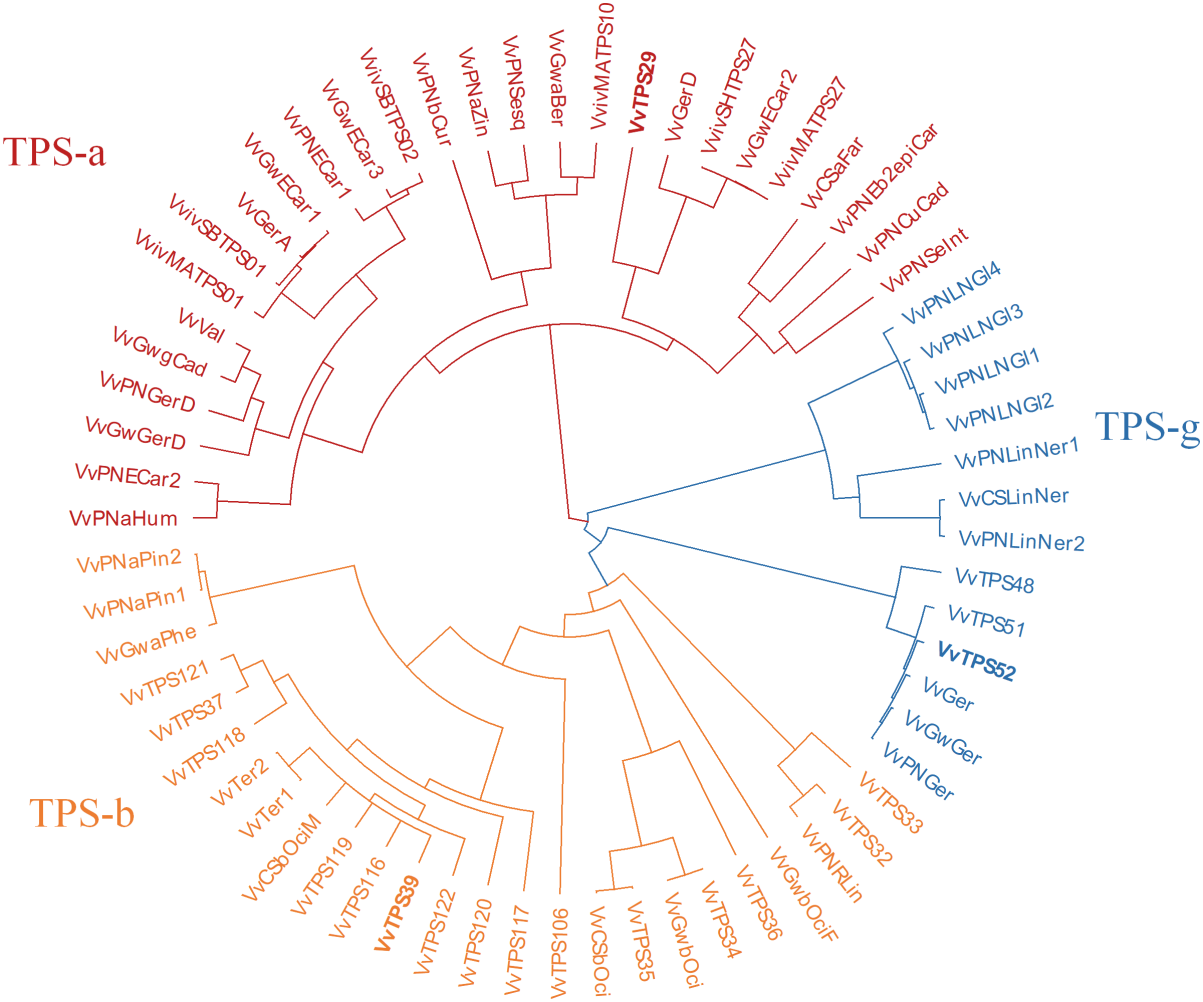
Phylogenetic tree of functionally characterised *V. vinifera* TPSs and VvTPSs identified in QTLs of interest from this study. The tree is coloured according to TPS-subfamilies: TPS-a subfamily is in red, TPS-b is orange and TPS-g subfamily is blue. Candidate genes are shown in bold typeface.

A phylogenetic tree comparing the *VvTPS*s in these QTL regions to functionally characterised *Vitis vinifera* TPSs is shown in **Figure 3**. VvTPS52 and VvTPS51 fall within a cluster with functionally characterised geraniol synthases (TPS-g), indeed *VvTPS52* is the PN40024 gene model for geraniol synthase (VvGer) (Martin et al., 2010) and therefore *VvTPS52*/*VvGer* was selected as candidate gene for the QTL associated with geraniol in this mapping population. VvTPS39, VvTPS119 and VvTPS116 cluster with VvTer1 and VvTer2, α-terpineol synthases in the TPS-a clade, however, *VvTPS119* and *VvTPS16* represent pseudogenes (disrupted by numerous deletions, frameshifts and/or stop codons) in the PN40024 v2 genome. *VvTPS39*/*VvTer* was therefore considered the likely candidate gene underpinning the QTL region. Lastly, VvTPS29 clusters with *trans*-caryophyllene synthases (VviSHTPS27, VviMATPS27 and VvGwECar2) and a germacrene D synthase (VvGerD) in the TPS-a clade.

### A SNP in *VvDXS1* is associated with monoterpene accumulation

**Figure 4:**
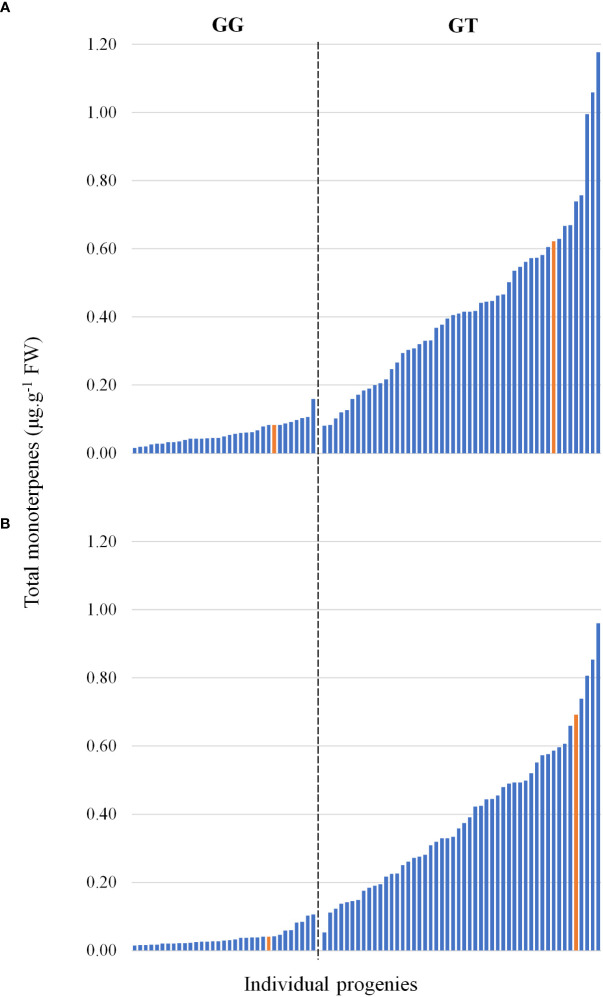
The total monoterpene content in the mapping progeny at **(A)** pre-veraison and **(B)** veraison. Each bar represents a member of the progeny, and the parents are shown in yellow. The bars are grouped into absence/presence of the *VvDXS1* SNP.

DNA sequencing of the *VvDXS1* alleles revealed that while ‘Deckrot’ was homozygous (GG) for the wild-type allele, G1-7720 was heterozygous (GT) for the muscat aroma linked SNP (Doligez et al., 2006; Battilana et al., 2009; Emanuelli et al., 2010). A TaqMan assay was used to ascertain how the mapping population under investigation segregates for the G>T SNP. Results show that of the 82 progenies genotyped, 50 were heterozygous (GT) and 32 were homozygous (GG) (**Table S7**). Comparison of the total monoterpene content in the progeny with the occurrence of the G>T SNP found that GG-progeny displayed relatively low monoterpene accumulation, while GT-progeny displayed relatively increased monoterpene accumulation levels, with a continuous variation in distribution (**Figure 4**). Orthogonal partial least squares (OPLS) analysis of the full volatile dataset across the population and *VvDXS1* genotypes show that the population clearly segregates for the SNP (**Figure S4**). The population segregate for the SNP across principal component 1 (PC1) which explains 27.1% of the variation.

### Expression of *VvTer* shows correlation with cyclic monoterpene accumulation

**Figure 5:**
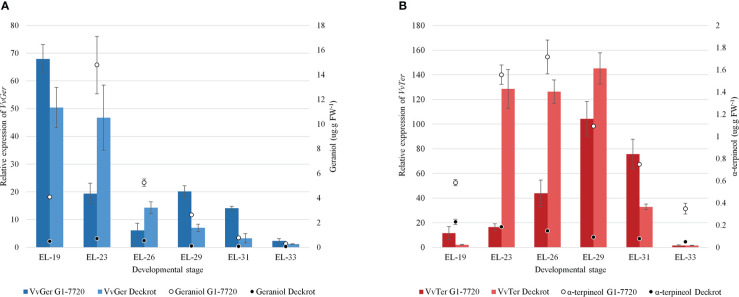
The expression of **(A)** *VvGer* and **(B)** *VvTer* in the parental cultivars, ‘Deckrot’ and G1-7720, during early berry development. The a-terpineol and geraniol concentrations at each stage is shown (values on right-side y-axis). n = 3; error bars represent standard error.

*VvTer* and *VvGer* gene expression was quantified during the late flower and early berry development in ‘Deckrot’ and G1-7720 (**Figure 5**). *VvGer* showed the highest level of expression in young flowers for both parents, while geraniol content only peaked in the following stage. *VvTer* expression was highest after fruit-set (EL-29), while α-terpineol concentration peaked earlier in the flowering stages. Both *VvTer* and *VvGer* expression, as well as α-terpineol and geraniol concentration, d-ecrease towards later berry development and is lowest in green berries (EL-33). Furthermore, α-terpineol and geraniol concentration is significantly higher in G1-7720 than ‘Deckrot’, irrespective of developmental stage or gene expression (**Figure 5**).

**Figure 6:**
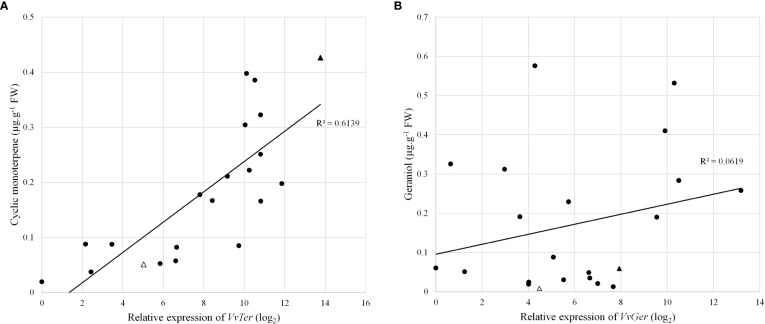
**(A)** *VvTer* expression compared to cyclic monoterpene content and **(B)** *VvGer* expression compared to geraniol content. Gene expression analysis and volatile quantification was done using pre-veraison berry skins. Circles represent progeny with the VvDXS1 SNP (GT), triangles represent the parents 'Deckrot' (empty) and G1-7720 (filled).

The expression of *VvTer* and *VvGer* was measured in a subset of the progeny to determine whether gene expression correlates with cyclic monoterpene or geraniol accumulation, respectively. The expression was measured in progenies with the *VvDXS1* SNP (GT) and without the *VvDXS1* SNP (GG). Linear regression analysis showed weak correlations between *VvTer* expression and cyclic monoterpene accumulation and between *VvGer* expression and geraniol accumulation (**Figure S5**). However, in progeny possessing the *VvDXS1* SNP, *VvTer* gene expression and cyclic monoterpene content showed relatively strong correlation (R = 0.78, p < 0.0001), indicating a positive linear relationship between *VvTer* expression and cyclic monoterpene accumulation (**Figure 6**).

### Multiple *VvTer* and *VvGer* gene copies were isolated from ‘Deckrot’ and G1-7720

**Figure 7:**
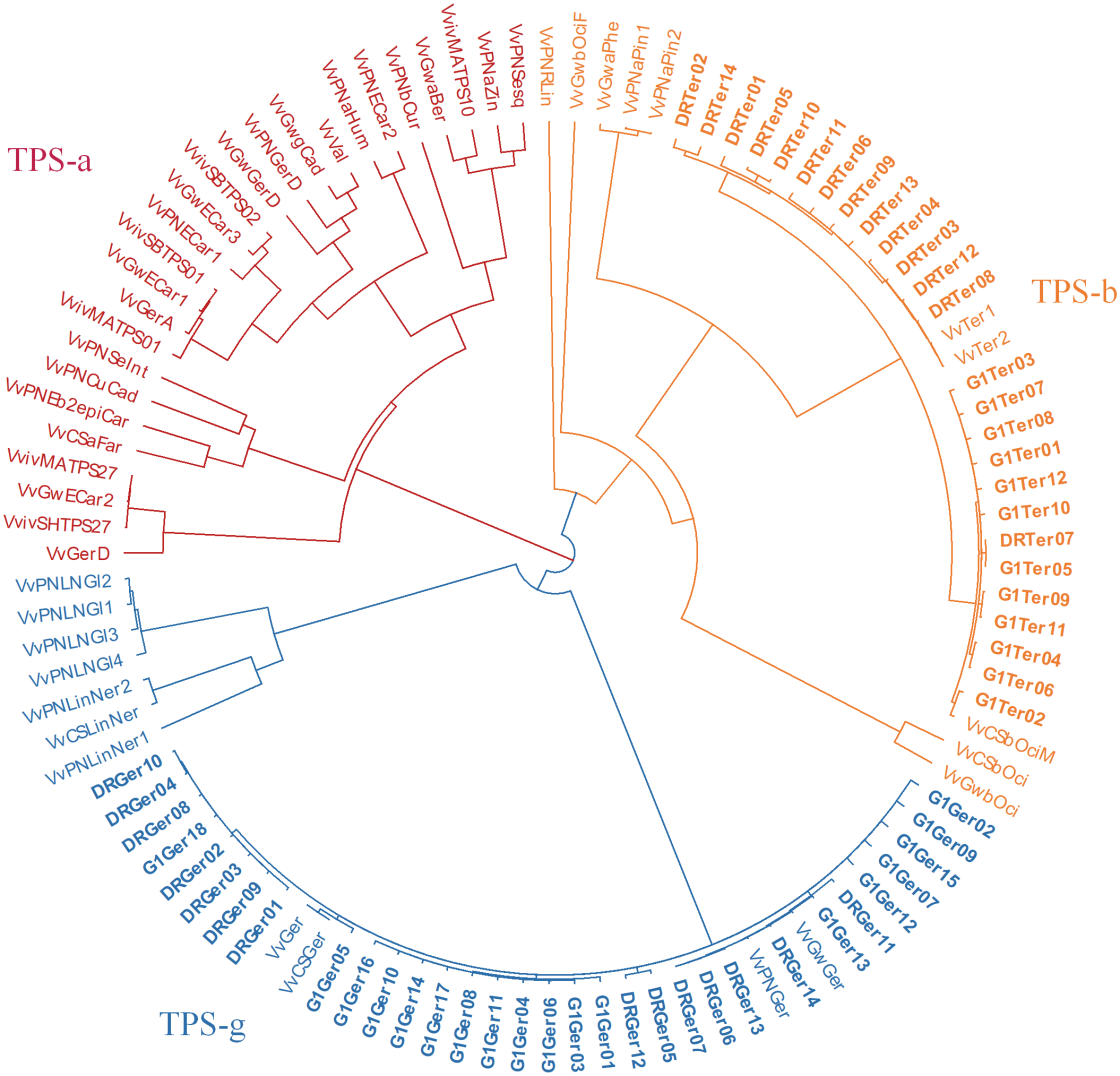
Phylogenetic tree (nucleotide sequences of coding regions) of ‘Deckrot’ and G1-7720’s *VvTer* and *VvGer* gene copies compared to functionally characterised *V. vinifera* terpene synthase genes. Clusters are coloured according to which TPS-subfamily the genes fall under. “G1” and “DR” indicate cultivar origin of isolated gene (G1-7720 and ‘Deckrot’, respectively).

Multiple cDNA clones of *VvTer* and *VvGer* from both ‘Deckrot’ and G1-7720 were sequenced in order to identify potential sequence variants. Using single primer pairs for each gene several unique expressed *VvTer* and *VvGer* gene copies were isolated from each parent cultivar (**Table 2** and **Figure S6**). The nucleotide sequences of all gene copies were compared to functionally characterised terpene synthases in *Vitis vinifera via* molecular phylogeny (**Table S8** and **Figure 7**). Regardless of cultivar, all *VvTer* sequences fall within the TPS-b family cluster while *VvGer* falls within the TPS-g subfamily. Interestingly, ‘Deckrot’ *VvTer* genes (*DRTer*) and G1-7720 *VvTer* genes (*G1Ter*) form two distinct clusters. Furthermore, *VvGer* gene copies from ‘Deckrot’ and G1-7720 share sequence similarity with functionally characterised *V. vinifera* geraniol synthases (*VvGer, VvGwGer* and *VvPNGer*), respectively. *DRTer* gene copies are similar to functionally characterised α-terpineol synthases (*VvTer1* and *VvTer2*), while *G1Ter* gene copies share sequence similarity with an (*E*)-β-ocimene/myrcene synthase (*VvCSbOciM*). *VvTer1, VvTer2* and *VvCSbOciM* are described as TPS39 genes with *VvTer1* and *VvTer2* being isolated from ‘Gewürtztraminer’, while *VvCSbOciM* was isolated from Cabernet Sauvignon (Martin and Bohlmann, 2004; Martin et al., 2010).

**Table 2:** The number of unique *VvTer* and *VvGer* cDNA clones that were identified for ‘Deckrot’ and G1-7720.

Parent cultivar	Gene of interest	cDNA clones sequenced	unique cDNA sequences	Putatively functional
‘Deckrot’	*VvTer*	16	14	10
	*VvGer*	16	14	12
G1-7720	*VvTer*	14	12	9
	*VvGer*	23	18	15

### Copy number of *VvTer* is correlated with cyclic monoterpene content

The relative copy numbers of *VvTer* and *VvGer* were determined for 82 progenies of the mapping population, as well as the parents (**Table S9**). Both *VvTer* and *VvGer* copy numbers were higher in G1-7720 than in ‘Deckrot’; *VvGer* had a 10:19 ratio for ‘Deckrot’:G1-7720 while *VvTer* had a 5:12 ratio. Gene copy number in the population were expressed as relative to ‘Deckrot’ gene copies, such that *VvTer* copy number ranges between 0.7-2.8 relative copies, while *VvGer* ranges between 1 – 2.1 relative copies. Additionally, a few individuals from the progeny were outliers. *VvGer* had the most outliers (6 progenies) with copy numbers up to 12.8 times higher than that observed for ‘Deckrot’.

**Figure 8:**
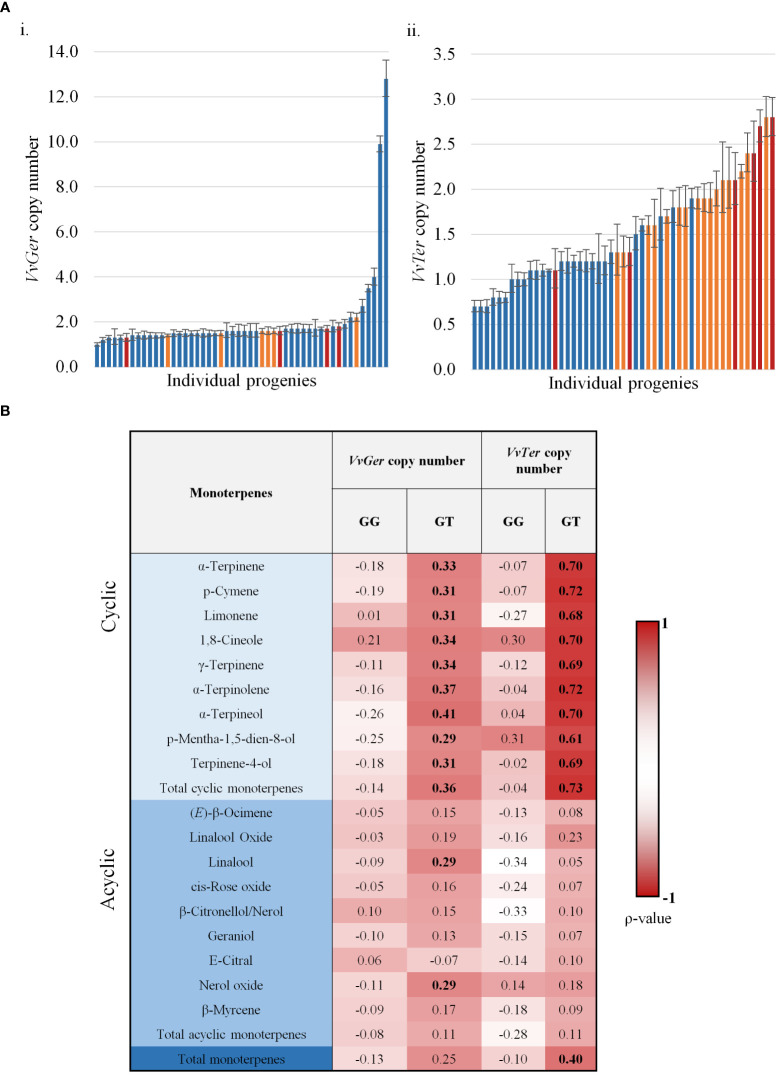
**(A)** The copy number of *VvGer *(i) and *VvTer* (ii) in progenies with the VvDXS1 SNP. Progenies were binned for low (blue), moderate (yellow) and high (red) geraniol (i) or total cyclic monoterpene (ii) content. n = 3; error bars represent standard error. **(B)** Spearman’s correlation between relative gene copy number *(VvGer and VvTer)* and monoterpenes. Correlation between gene copy number and monoterpenes were analysed separately for progeny with the *VvDXS1* SNP (GT) and without the SNP (GG). Correlation values in boldface were statistically significant; p < 0.001.

Spearman’s rank correlation (ρ) analysis found *VvTer* copy number possessed a strong correlation with *VvTer* expression (ρ = 0.748), but *VvGer* copy number and expression had weak correlation (ρ = 0.189). The geraniol and total cyclic monoterpene content was binned into three categories: low, moderate and high (**Figure 8A**). *VvGer* copy number shows no discernible pattern between geraniol content and copy number, while total cyclic monoterpene content seems to show a positive correlation with *VvTer* copy number. The correlation between gene copy number and several monoterpenes was investigated in the progeny (**Figure 8B**). There was no correlation between *VvGer* copy number and any monoterpenes in the progeny, regardless of the presence of the *VvDXS1* SNP. However, *VvTer* showed different degrees of correlation with cyclic monoterpenes depending on the presence of the *VvDXS1* SNP. Progeny which did not have the G>T *VvDXS1* SNP (GG) showed weak correlation (-0.1< ρ < 0.1) between *VvTer* copy number and cyclic monoterpenes, while in progeny with the *VvDXS1* SNP (GT) there was a strong positive correlation (ρ > 0.6).

### Heterozygous distribution of terpene synthases observed in a diploid *Vitis vinifera* genome

**Figure 9:**
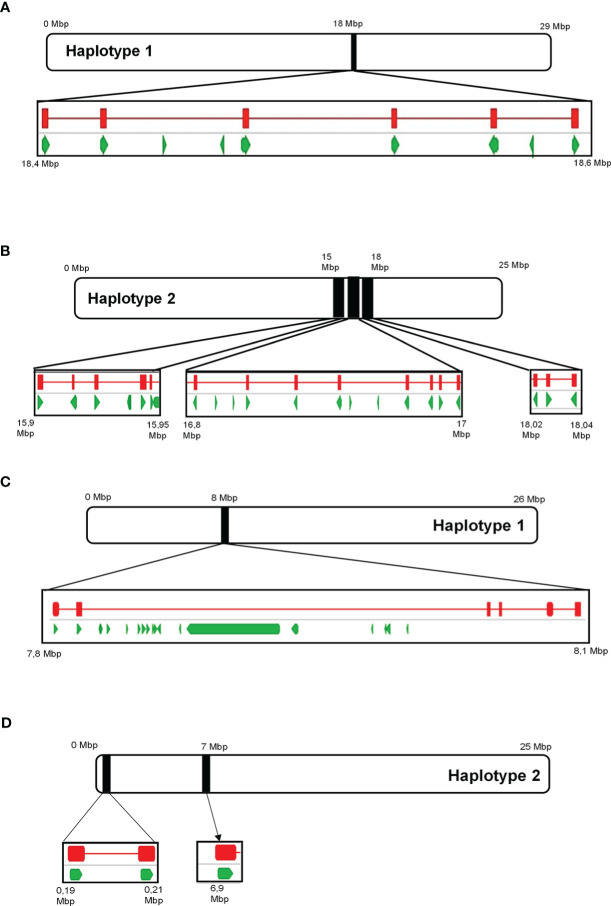
The position of predicted ‘Cabernet Sauvignon’ *VvTer* and *VvGer* genes on chromosome 13 **(A, B)** and chromosome 12 **(C, D)**, respectively. Black blocks indicate the position of the predicted *VvTer* or *VvGer* clusters on the chromosomes, and squares underneath provide a magnified view of the region. Green arrows represent predicted genes as annotated in Massonnet et al. (2020), while red lines indicate the position of BLAST hits for *VvTer* or *VvGer*.

Analysis of the draft diploid genome of Cabernet Sauvignon identified 29 putative α-terpineol synthase (*VvCSTer*) loci, and nine putative geraniol synthase (*VvCSGer*) loci (**Table S10**). The majority of α-terpineol synthases were localised to chromosome 13 while geraniol synthases predominantly localised to chromosome 12, and the remaining genes were unplaced (**Table S10**). The nucleotide coding sequences of *VvCSTer* genes had a pairwise identity of 90.8% and *VvCSGer* genes shared 98.2% pairwise identity. Furthermore, the loci appeared in clusters on the chromosomes, with adjacent genes in clusters being less than 100 kb apart (**Figure 9**). Haplotype 2 of chromosome 13 has three separate *VvCSTer* clusters which are approximately 1 Mb apart. The high similarity as well as proximity of these genes indicate an extensive level of tandem gene duplication, particularly for *VvCSTer* genes.

11 of the 29 predicted Cabernet Sauvignon α-terpineol synthase loci encode for putatively functional enzymes, while 4 of the 9 geraniol synthase loci encode for putatively functional enzymes. A phylogenetic tree (**Figure S7**) was constructed to compare the putatively functional Cabernet Sauvignon α-terpineol and geraniol synthases with functionally characterised *Vitis vinifera* terpene synthases. All putatively functional VvCSGer proteins fall within the TPS-g cluster while VvCSTer proteins fall within the TPS-b cluster.

## Discussion

### Elucidation of the genetics underlying the VOC profile of the mapping population

Studies analysing volatile accumulation show mixed results with regards to the accumulation pattern of different VOC classes (Ferrandino et al., 2012; Vilanova et al., 2012; JI et al., 2021; Liu et al., 2022), and it is therefore challenging to compare the metabolite data observed here with previous studies. Importantly, however, the genetic control of VOC accumulation was evident through the observed segregation of compound accumulation in the population under study, and the subsequent associated QTLs that were identified. The significant QTL on chromosome 5 associated with total monoterpene content, houses the well described *VvDXS1* gene (Doligez et al., 2006; Battilana et al., 2009; Emanuelli et al., 2010). In the population under investigation here we show that the SNP contributing to high terpene level and a “muscat like aroma” is derived from G1-7720, the aromatic parent. Importantly this SNP was found to be a prerequisite for high monoterpene levels in the progeny, but not an absolute indicator (**Figures 4**, **S4**). This is likely due to the multigenic nature of terpene formation, further highlighted by the various other QTLs identified in this study and others (Doligez et al., 2006; Battilana et al., 2009; O’Reilly-Wapstra et al., 2011; Yu et al., 2017; Reichardt et al., 2020; Barbey et al., 2021). Importantly, accumulation of the plastid derived C13-norisoprenoids also associated with this SNP, while the cytosolic derived sesquiterpenes did not. This suggests that, at least in the case of this grapevine population, there is limited isoprenoid crosstalk from plastid to cytosol (Gutensohn et al., 2013).

While the *VvDXS1* SNP can be considered a master regulator of monoterpene accumulation, candidate genes which co-localise with QTLs for geraniol, cyclic monoterpenes and sesquiterpenes were also identified in this study. Geraniol shows a QTL on chromosome 12 which co-localised with a cluster of eight terpene synthase genes. *VvTPS52*, one of the eight VvTPSs in this region, was predicted to function as a geraniol synthase (*VvGer*) and has indeed been previously functionally characterised as a geraniol synthase in various other cultivars (Martin et al., 2010). Several cyclic monoterpenes showed overlapping associated QTLs on chromosome 13. These QTLs co-localise with a cluster of terpene synthase genes containing putative α-terpineol synthases. *VvTPS39* was proposed as candidate gene for this QTL as it was the closest related gene that was not a predicted pseudogene and has been characterised as an α-terpineol synthase (*VvTer*) *via in vitro* enzyme assay in ‘Gewurztraminer’ (Martin and Bohlmann, 2004). Interestingly, the authors found that VvTPS39 produced α-terpineol, 1,8-cineole and β-pinene as major products and various minor product including α-thujene, α-pinene, myrcene, sabinene, β-pinene, limonene, and terpinolene. The multi-product nature of terpene synthases may explain why this study found multiple cyclic monoterpenes mapping to the same region on chromosome 13. It is likely that *VvTer* (and possibly some of the other *VvTPSs* that clustered in the same region) produce cyclic monoterpenes in varying ratios, with α-terpineol being the major product.

The sesquiterpenes α-cubebene and trans-caryophyllene, associated with a large QTL (approximately 11.65 Mbp) on chrosomome 19. *VvTPS29* was proposed as a candidate gene and this was further supported by molecular phylogenetic evidence. VvTPS29 showed to be closely related to functionally characterised trans-caryophyllene synthases and a germacrene D synthase (**Figure 3**). Additionally, an isocaryophyllene/β-cadinene synthase (VvShirazTPS-Y2) which shares 100% similarity with VvTPS29 has been characterised in ‘Shiraz’ (Dueholm et al., 2019).

Non-terpene VOCs were also found to associate with QTLs in this study. C6-compounds or green leaf volatiles (GLVs) were the most abundant VOCs present in the progeny at all developmental stages, as has been reported in other studies (Kalua and Boss, 2009; Vilanova et al., 2012). A QTL associated with trans-2-hexenal and total aldehydes was identified on chromosome 2 and co-localised with a Stearoyl-[acyl-carrier-protein] 9-desaturase 6 (SAD). SAD catalyses the conversion of stearoyl-ACP to oleoyl-ACP which serves as a precursor for the fatty acids α-linolenic acid and linoleic acid. α-linolenic acid and linoleic acid in turn can be broken down to form green leaf volatiles which are synthesised *via* the lipoxygenase (LOX) pathway (Matsui and Engelberth, 2022). GLVs are rapidly released upon plant wounding which is thought to play a role in plant signalling and defence (Matsui, 2006; Bouwmeester et al., 2019; Matsui and Engelberth, 2022). C6-aldehydes and -alcohols contribute to the undesirable “green aroma” of wine (Dunlevy et al., 2009), and therefore understanding their metabolism may contribute to improved wine aroma.

A QTL associated with cis-rose oxide, E-citral and nerol oxide was identified in the same region on chromosome 17. A cluster of five short chain dehydrogenase/reductases which co-localised with the QTL were selected as candidate genes. All three of the compounds are derived from geraniol, however importantly geraniol did not associate with this QTL. Geraniol dehydrogenases which catalyse the formation of citral and citronellol have been identified in several plant species but not in grapevine (Iijima et al., 2006; Sato-Masumoto and Ito, 2014; Xu et al., 2017). Lin et al. (2019) summarises a proposed pathway for citronellol, nerol and cis-rose oxide in grapevine but to date none of the proposed enzymes in the pathway have been identified and characterised. Further characterisation of the *VvSDR*s identified in this QTL may therefore prove promising in elucidating the biosynthesis of these compounds.

### Investigation of the *VvTer* and *VvGer* loci highlights extensive gene duplication

In an attempt to isolate the monoterpene synthase genes underlying the QTLs associated with geraniol and cyclic monoterpene accumulation it was discovered that there were more gene copies than represented in the reference genome. Furthermore, as the genes isolated represent cDNA clones, the number of gene copies shown in **Table 2** is not exhaustive representation of all the possible *VvTer* and *VvGer* copies. Importantly, phylogenetic analysis of the sequenced ‘Deckrot’ and G1-7720 *VvTer* and *VvGer* gene copies found strong cultivar specific clustering, indicating this gene duplication has occurred post cultivar diversification.

Due to the somewhat surprising nature of observed *VvTPS* gene duplication in the cultivars in this study an *in silico* analysis of *VvTer* and *VvGer* in the diploid ‘Cabernet Sauvignon’ genome was performed. This analysis confirmed massive gene duplication and revealed that both genes occur in tandem duplications on chromosome 13 and chromosome 12, respectively. *VvTer* had the most extensive duplications with 29 copies on the ‘Cabernet Sauvignon’ genome. Analysis of the draft diploid genome further indicated that *VvTer* genes were unequally distributed between the two haplotypes of chromosome 13. *VvGer* genes also occurred unequally distributed on the haplotypes of chromosome 12. The distribution *VvTer* and *VvGer* loci were uneven on the two haplotypes of chromosomes 12 and 13, revealing a high level of hemizygosity. Furthermore, due to the high sequence similarity between these genes, it is difficult to predict which loci are allelic and which are hemizygous.

Further study is necessary to ascertain the function of the *VvTPS* candidate genes in this mapping population. High levels of gene duplication contribute to the diverse functions of TPSs through neofunctionalisation and sub-functionalisation (Tholl, 2015). Furthermore, the function of TPSs can be changed by small amino acid substitutions which alter the active site conformation and further contributes to the diverse functions of TPSs (Degenhardt et al., 2009; Karunanithi and Zerbe, 2019) and this has also been shown by the presence of cultivar-specific TPSs in grapevine (Martin et al., 2010; Drew et al., 2016; Smit et al., 2019) therefore it remains a challenge to predict the function of *VvTPSs* based on sequence similarity to functionally characterised genes alone.

### *VvTer* gene copy number and expression correlate with cyclic monoterpene accumulation

The pattern of *VvGer* and *VvTer* expression was similar in both parent cultivars during berry development, but the expression patterns did not correspond with the accumulation of α-terpineol or geraniol. Previous studies have shown that *VvTPS* developmental expression patterns do not always match the accumulation patterns of their associated metabolites (Martin et al., 2012; Matarese et al., 2013; Matarese et al., 2014). Furthermore, only the volatile fractions of geraniol and α-terpineol were quantified in this study, while monoterpenes are also present in non-volatile glycosylated forms in grapevine (Dunlevy et al., 2009; Hjelmeland and Ebeler, 2015).

However, the expression of *VvTer* across the mapping population correlated with several cyclic monoterpenes, specifically in progeny containing the *VvDXS1* SNP. The high similarity between *VvTer* paralogs potentially skews qPCR data as the primers have the potential to bind to more than one *VvTer* gene locus. This fact together with the *VvTPS* gene duplication data described above led to investigation of the *VvTer* gene copy number. Importantly, the parent cultivars under investigation showed unequal *VvTer* gene copy number, with G1-7720 showing 2.4-fold greater number of copies of *VvTer* than ‘Deckrot’. Relative *VvTer* gene copy number showed a positive correlation with the accumulation of several individual cyclic monoterpenes as well as total cyclic monoterpenes. The correlation of *VvTer* gene expression with multiple monoterpenes is in agreement with the fact that this gene cluster co-localises with the QTL for cyclic monoterpenes. Furthermore, *VvTer* is potentially a multi-product forming enzyme similar to other terpene synthases (Martin et al., 2010; Karunanithi and Zerbe, 2019), and potentially forms several structurally related monoterpene compounds, such as cyclic monoterpenes.

Hypotheses for the correlation of *VvTer* copy number and cyclic monoterpene accumulation are somewhat more speculative and are detailed in the following sections. The first hypothesis is that *VvTer* copy number may cause a dosage effect, i.e. the more copies of the gene, the more transcripts produced, and the more enzyme translated. This is supported by the strong correlation between *VvTer* expression and copy number. However, dosage does not explain why some low copy number individuals have high levels of cyclic monoterpenes and vice versa (**Figure 8**).

**Figure 10:**
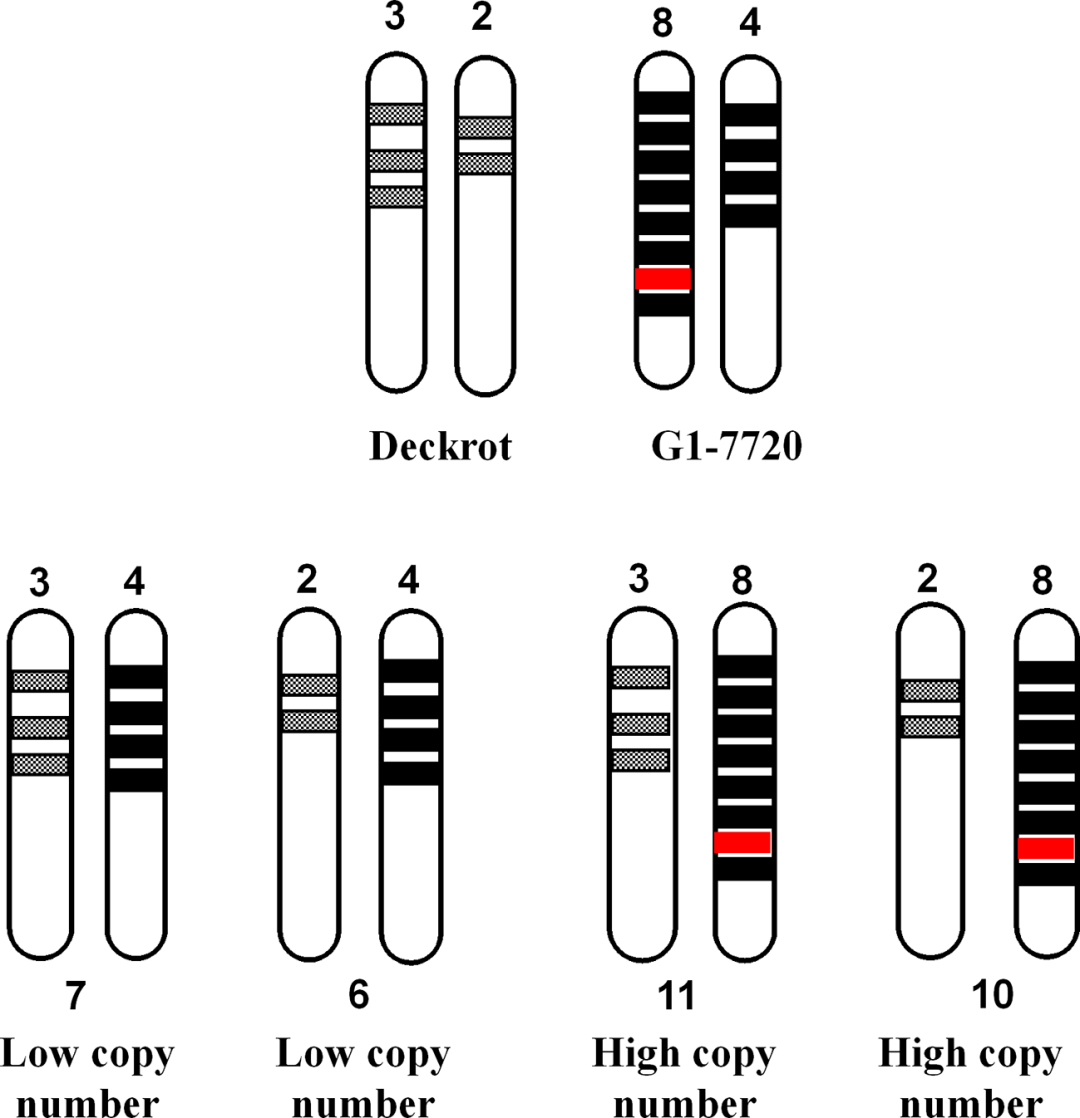
A diagram to explain the correlation between *VvTer* copy number and cyclic monoterpene accumulation. *VvTer* genes are represented by shaded blocks and the “hyper-functional” gene is shown in red. The “hyper-functional” gene falls on the parent allele with the highest copy number and is the causal factor for higher cyclic monoterpene content in this parent. The progeny from ‘Deckrot’ x G1-7720 have an equal chance having one of four possible *VvTer* copies: 6, 7, 10 or 11. Progenies with 6 or 7 copies would be considered “low copy number” individuals and would not inherit the “hyper-functional” gene resulting in lower/normal accumulation of cyclic monoterpenes. Progenies with 10 or 11 copies would be considered “high copy number individuals” and will inherit the “hyper-functional” gene resulting in higher cyclic monoterpene accumulation.

Another possible explanation is that a “hyper-functional” gene/genetic element co-localises with the high copy number allele (**Figure 10**). “Hyper-functional” is defined as any sequence variation which significantly improves cyclic monoterpene synthase enzyme activity, resulting in increased cyclic monoterpene biosynthesis. In **Figure 10** we assume that ‘‘Deckrot’ has 5 copies of *VvTer* (3 copies on allele A and 2 copies on allele B) and G1-7720 has 12 copies (8 copies on allele A and 4 copies on allele B) as this fulfils the 5:12 (‘Deckrot’:G1-7720) ratio of *VvTer* copy number in the parents. It is important to note that while the exact gene copy numbers cannot be determined, sequencing of cDNA clones suggests a relatively high *VvTer* copy number. Furthermore, the fact that the progeny typically presented a higher copy number than ‘Deckrot’, and a lower copy number than G1-7720, indicate that both alleles for G1-7720 have a higher copy number than the ‘Deckrot’ alleles.

In this hypothesis the progeny has an equal chance of inheriting one allele from each parent in four potential different combinations. If a hyper-functional gene/genetic element co-localizes with the high copy number allele, all individuals that inherited the high copy number allele will have high cyclic monoterpene levels. Therefore, individuals with a high copy number do not have higher levels of cyclic terpenes due to additive effects but due to the co-segregation of high copy number and a “gain-of-function” gene/genetic element. The high level of hemizygosity of *VvTer* genes in the ‘Cabernet Sauvignon’ genome, as well as the large discrepancy between *VvTer* copy numbers of ‘Deckrot’ and G1-7720 (G1-7720 has 2.4 times as many *VvTer* copies as ‘Deckrot’) support this theory. While the exact distribution of *VvTer* loci in the parent cultivars are unknown, it is safe to assume that both also have high hemizygosity which results in them having either a “high copy number” or “low copy number” allele.

This hypothesis also explains why some individuals have a low copy number but high cyclic monoterpene level. If the hyper-functional element is transferred to a “low copy number” allele during a recombination event, some low copy number individuals will have high cyclic monoterpene levels. Recombination events at a specific locus are rare and would thus only affect a few individuals in the population (Choi, 2017). Furthermore, this hypothesis explains why there is no correlation between *VvGer* copy number and geraniol levels. The difference in *VvGer* copy numbers between G1-7720 and ‘Deckrot’ is less prominent, therefore making differentiation between “high copy number” and “low copy number” alleles difficult. The “hyper-functional” element of *VvGer* thus co-segregates with high copy number similarly to *VvTer*, but the difference between high and low copy number is too small to make any significant distinction and no correlation is observed.

## Conclusion

During berry development *VvGer* and *VvTer* gene expression patterns did not align with the accumulation patterns of geraniol and α-terpineol, respectively, indicating that terpene synthase gene expression cannot solely be used to infer gene function. Furthermore, the extensively duplicated nature of the TPS gene family complicates gene expression analysis as well as identification of sequence variants leading to enzyme activity modulation. *VvGer* and *VvTer* genes are present in tandemly duplicated gene clusters and these clusters are unequally distributed across chromosome haplotypes indicating extreme levels of hemizygosity for these genes. Furthermore, *VvTer* is highly duplicated in grapevine genomes and *VvTer* copy number correlated with cyclic monoterpene accumulation.

Extensive gene duplication is a common feature of TPS-gene families in several plant species and results in the presence of multiple highly similar gene paralogs in these genomes. While the influence of small polymorphisms, such as SNPs and indels, on TPS functions and terpene accumulation are commonly researched, very few studies investigate the influence of this gene copy number variation on terpene accumulation. The knowledge gained from this study further contributes to our understanding of terpene metabolism in grapevine and the potential impact of *TPS* copy number variation on grapevine terpene accumulation.

## Data availability statement

The original contributions presented in the study are included in the article/**Supplementary materials**. Further inquiries can be directed to the corresponding author.

## Author contributions

RB designed and performed experiments, analyzed data, and was the primary author of the manuscript. JV performed experiments, analyzed data and co-wrote the manuscript. DN performed experiments. PB designed and performed experiments. JL conceptualized the research, designed experiments, analyzed data, and co-wrote the manuscript. All authors contributed to the article and approved the submitted version.
