# Supplementary material for: Grapevine genome analysis demonstrates the role of gene copy number variation in the formation of monoterpenes
Source: Front Plant Sci. 2023 Mar 16;14:1112214. doi: 10.3389/fpls.2023.1112214 (PMC10061021; doi:10.3389/fpls.2023.1112214)
Supplement: Supplementary file 1 [file DataSheet_1.pdf]

Supplementary Figures

Grapevine genome analysis demonstrates the role of gene copy number variation in the formation of monoterpenes

Robin Nicole Bosman[1], Jessica Anne-Marie Vervalle[2], Danielle Lisa Brown[1], Phyllis Burger[3], Justin Graham Lashbrooke[1]*

***Correspondence:**

Justin Graham Lashbrooke

jglash@sun.ac.za

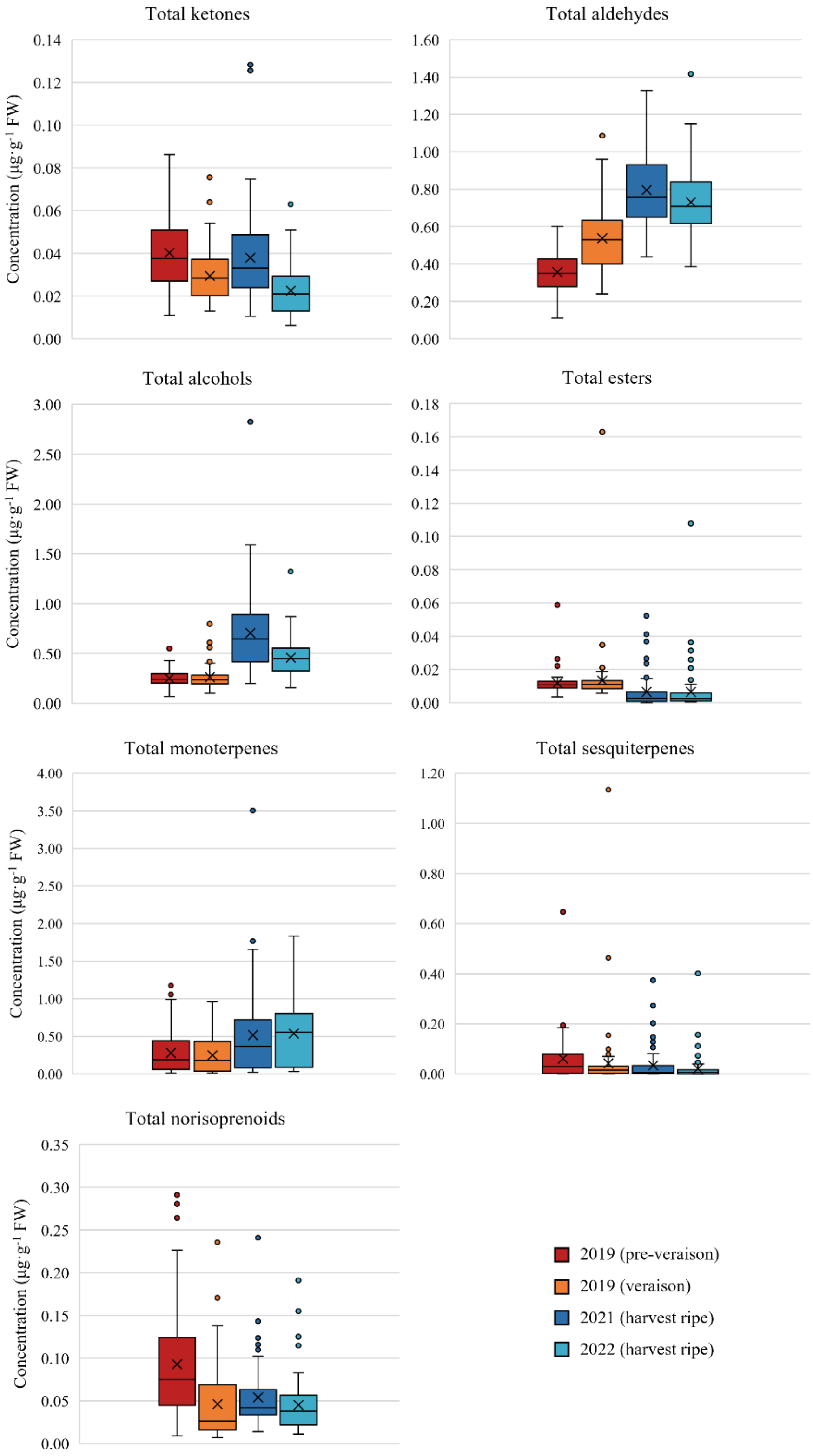

Supplementary Figure 1. The distribution of VOC compound classes in the mapping population identified in this study.

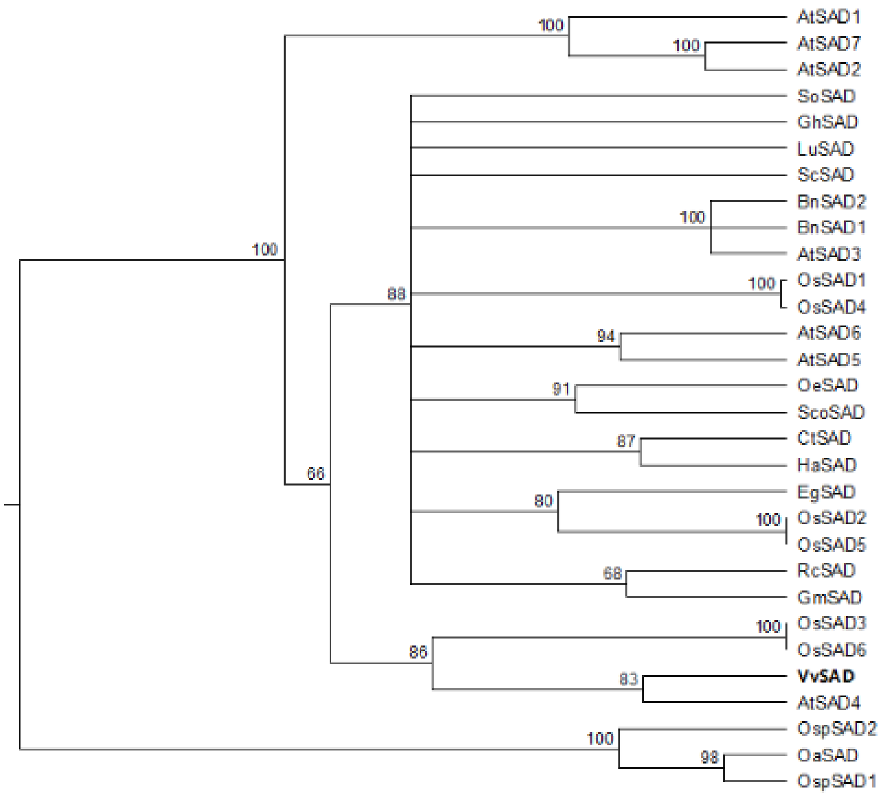

Supplementary Figure 2. Molecular phylogenetic tree of functionally characterised SADs and VvSAD (bold typeface).

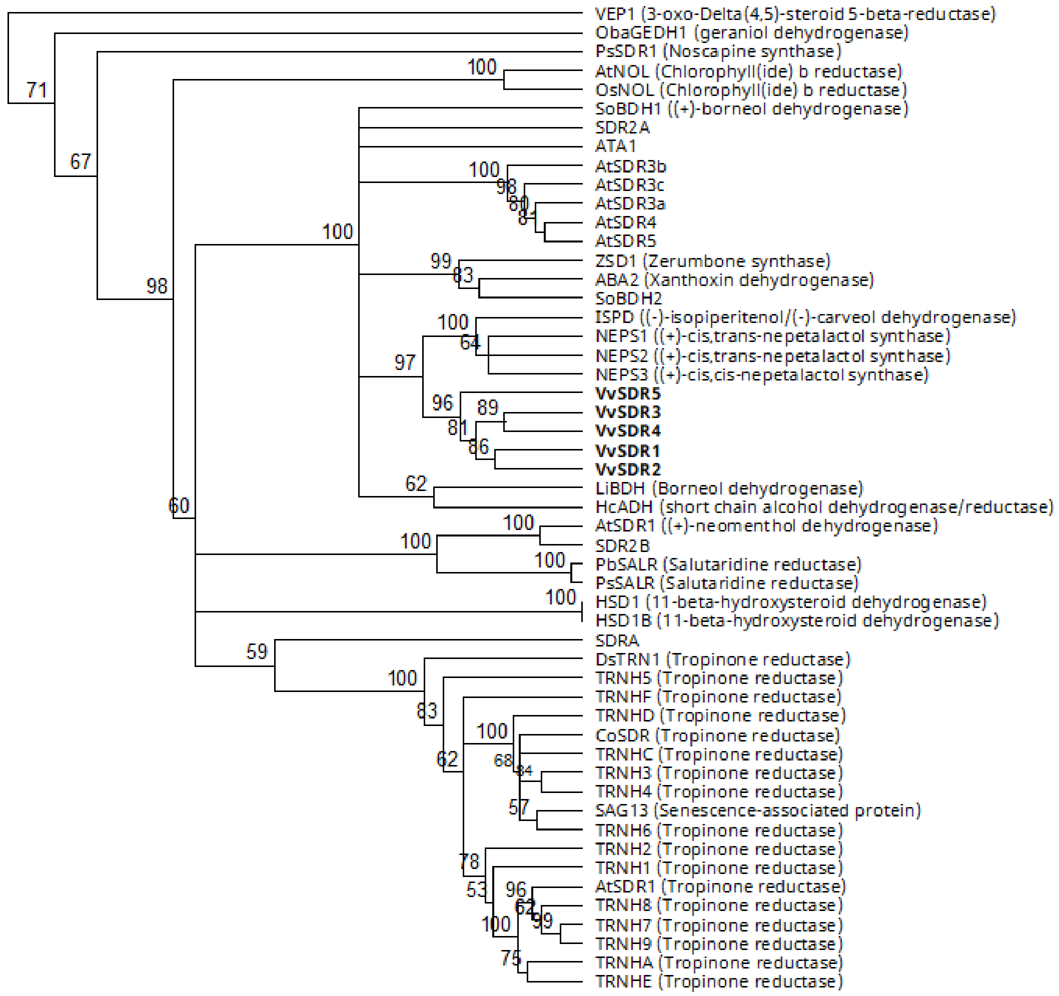

Supplementary Figure 3. Molecular phylogenetic tree of functionally characterised SDRs and VvSDRs (bold typeface). The UniProtKB/SwissProt annotated function of each SDR is shown in brackets (where available).

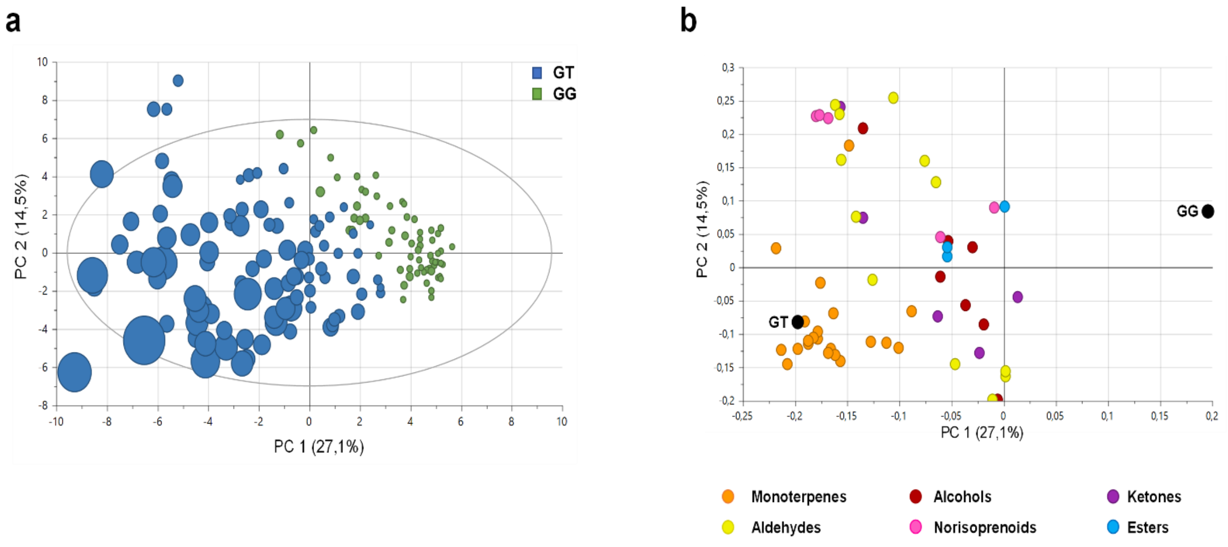

Supplementary Figure 4. **a)** OPLS of VOC dataset and *VvDXS1* genotypes. Data points are coloured according to the presence of the *VvDXS1* SNP: GT = heterozygous for the SNP and GG = wild-type. Data points are sized based on total monoterpene concentration; samples with higher monoterpene concentrations are bigger. **b)** Loading plot. Variable data points are coloured according to which compound class they fall within.

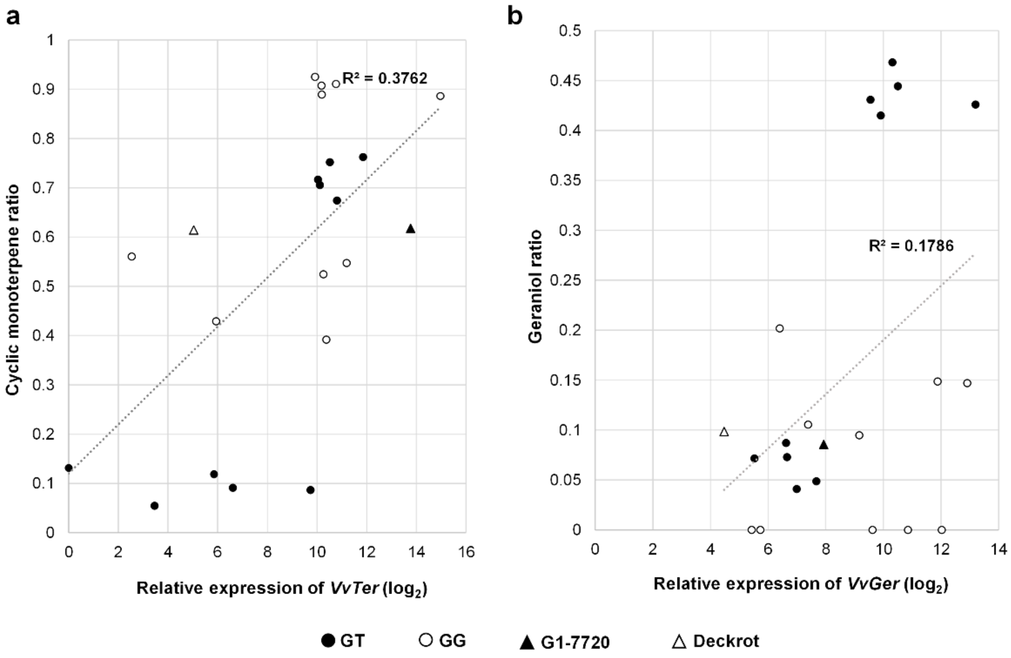

Supplementary Figure 5. a) VvTer expression plotted against cyclic monoterpene ratio and **b)** VvGer expression plotted against geraniol ratio. Each graphs represent a subset of 20 progenies from the mapping population which includes ten progenies with the VvDXS1 SNP and ten without. The subset is further divided by five progenies with the highest and five progenies with the lowest cyclic monoterpene ratio (a) and five progenies with highest and five progenies with the lowest geraniol ratio (b).

a

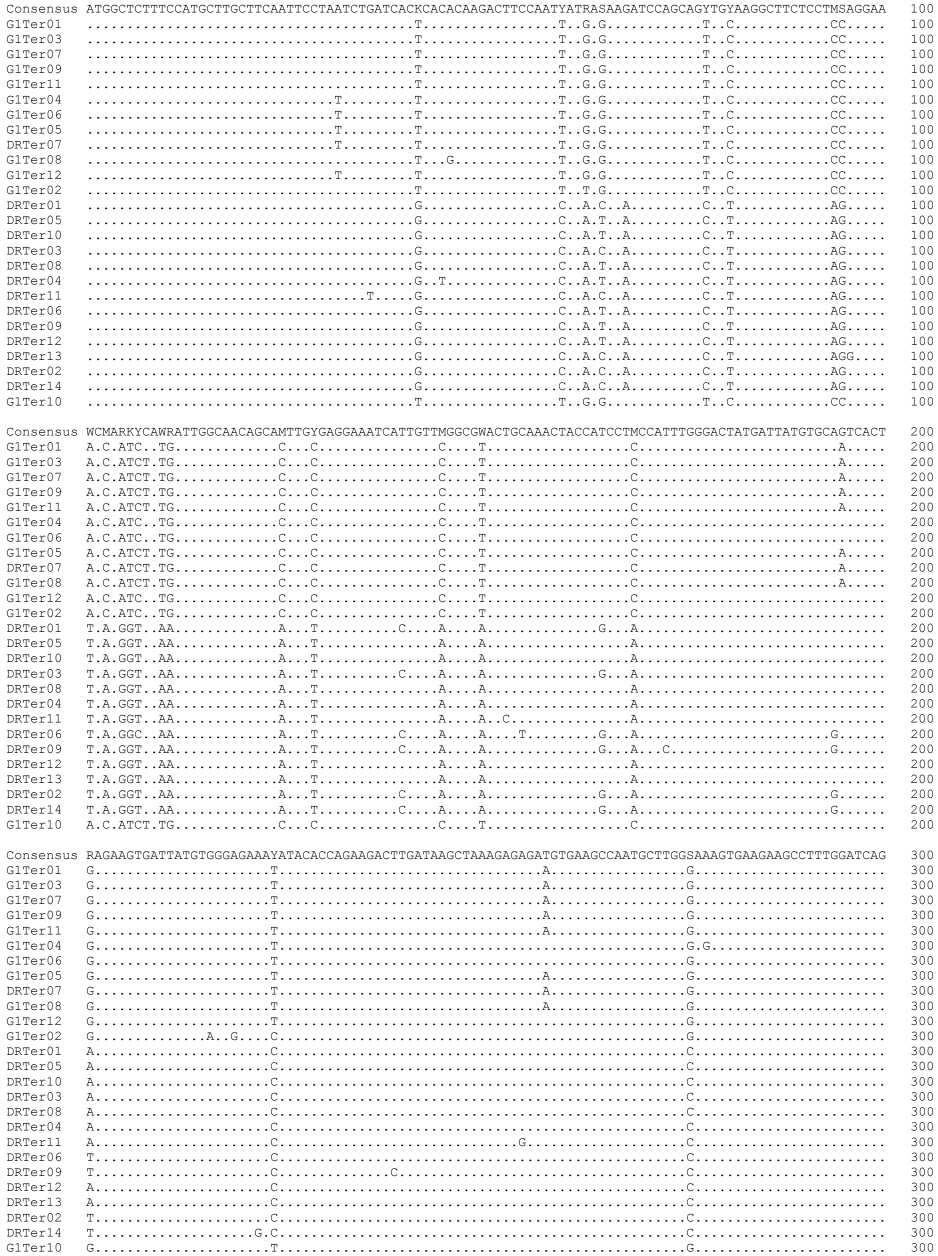

```
Consensus CTGGAGCTAATCGATGTYTTGCAAAGGCTTGGAMTATATTATCACTTCAAGGATGMAATAAAGAGAATATTGRACAGCATATACAACCAGTACAATCRGC   400
G1Ter01   .................T...............C.....................C................G........................A..   400
G1Ter03   .................T...............C.....................C................G........................A..   400
G1Ter07   .................T...............C..................G..C................G........................A..   400
G1Ter09   .................T...............C.....................C................G........................A..   400
G1Ter11   .................T...............C.....................C................G........................A..   400
G1Ter04   .................T...............C.....................C................G........................A..   400
G1Ter06   .................T...............C.....................C................G........................A..   400
G1Ter05   .................T...............C.....................C................G........................A..   400
DRTer07   .................T...............C.....................C................G........................A..   400
G1Ter08   .................T...............C.....................C................G........................A..   400
G1Ter12   .................T...............C.....................C................G........................A..   400
G1Ter02   .................T...............C.....................C................G........................A..   400
DRTer01   .................C...............A.....................A................A........................G..   400
DRTer05   .................C...............A.....................A................A........................G..   400
DRTer10   .................C...............A.....................A................A........................G..   400
DRTer03   .................C...............A.....................A................A........................G..   400
DRTer08   .................C...............A.....................A................A........................G..   400
DRTer04   .................C...............A.....................A.........G......A........................G..   400
DRTer11   ..........CA.....C...............A.....................A................A........................A..   400
DRTer06   .................C...............A.....................A................A........................G..   400
DRTer09   ......A..........C...............A.....................A................A........................G..   400
DRTer12   .................C...............A.....................A................A..............T.........G..   400
DRTer13   .................C...............A.....................A................A........................G..   400
DRTer02   .................C...............A.....................A................A........................G..   400
DRTer14   .................C...............A.....................A................A........................G..   400
G1Ter10   .................T...............C.....................C................G........................A..   400

Consensus AYGAAGAGTGGMAGAAAGATGATTTATACGCAACAGCTCTTGAATTTAGACTGTTAAGGCAGCATGGCTATGATGTGCCTSAAGATGTTTTTAGTAGATT   500
G1Ter01   .C.........A....................................................................G...................   500
G1Ter03   .C.........A....................................................................G...................   500
G1Ter07   .C.........A....................................................................G...................   500
G1Ter09   .C.........A....................................................................G...................   500
G1Ter11   .C.........A.....................................G..............................G...................   500
G1Ter04   .C.........A....................................................................G...................   500
G1Ter06   .C.........A....................................................................G...................   500
G1Ter05   .C.........A.....................................G..............................G...................   500
DRTer07   .C.........A.....................................G..............................G...................   500
G1Ter08   .C.........A.....................................G................A.............G...................   500
G1Ter12   .C.........A................................C...................................G...................   500
G1Ter02   .C.....C...A....................................................................G...................   500
DRTer01   .T.........C....................................................................C...................   500
DRTer05   .T.........C....................................................................C...................   500
DRTer10   .T.........C....................................................................C...................   500
DRTer03   .T.........C........................................................C...........C...................   500
DRTer08   .T.........C....................................................................C...................   500
DRTer04   .T.........C....................................................................C...................   500
DRTer11   .T.........C....................................................................C...................   500
DRTer06   .T.........C.................A..................................................C....C..............   500
DRTer09   .T.........C.................A..................................................C...................   500
DRTer12   .T.........C....................................................................C...................   500
DRTer13   .T.........C....................................................................C...................   500
DRTer02   .T.........C.................A..................................................C....C..............   500
DRTer14   .T.........C.................A..................................................C....C..............   500
G1Ter10   .C.........A....................................................................G...................   500

Consensus CAAGGACGAWACAGGGAGCTTTAAGGCATGCCTGTGTGAAGATATGAAGGGARTGCTCTGCTTGTACGAAGCTTCATACCTTTGTGTACAAGGAGAAAGT   600
G1Ter01   .........A.......T..................................G...............................................   600
G1Ter03   .........A.......T..................................G...............................................   600
G1Ter07   .........A.......T..................................G...............................................   600
G1Ter09   .........A.......T..................................G...............................................   600
G1Ter11   .........A.......T..................................G...............................................   600
G1Ter04   .........A.......T..................................G...............................................   600
G1Ter06   .........A.......T..................................G...............................................   600
G1Ter05   .........A..........................................G...............................................   600
DRTer07   .........A..........................................G...............................................   600
G1Ter08   .........A.......T..................................G...............................................   600
G1Ter12   .........A.......T..................................G...............................................   600
G1Ter02   .........G..........................................C...............................................   600
DRTer01   .........T..........................................A...............................................   600
DRTer05   .........T..........................................A...............................................   600
DRTer10   .........T..........................................A...............................................   600
DRTer03   .........T..........................................A...............................................   600
DRTer08   .........T..........................................A...............................................   600
DRTer04   .........T..........................................A...............................................   600
DRTer11   .........T..........................................A...............................................   600
DRTer06   .........T..........................................A............................C..................   600
DRTer09   .........T..........................................A...............................................   600
DRTer12   .........T..........................................A...............................................   600
DRTer13   .........T..........................................A...............................................   600
DRTer02   .........A...................................C......C.......T.....T..............A..................   600
DRTer14   .........A...................................C......C.......T.....T..............A..................   600
G1Ter10   .........A.......T..................................G...............................................   600

Consensus ACCWTGGAGCAGGCCAGAGAYTTCGCACACAGACATCTTGGAAAGGGCCTTGAGCACAACATAGATCAAAATCTTGCCATTGAGGTGAACCATGCCTTAG   700
G1Ter01   ...T................T...............................................................................   700
G1Ter03   ...T................T...............................................................................   700
G1Ter07   ...T................T...............................................................................   700
G1Ter09   ...T................T...............................................................................   700
G1Ter11   ...T................T............................C..................................................   700
G1Ter04   ...T................T...............................................................................   700
G1Ter06   ...T................T..............................A................................................   700
G1Ter05   ...T................T...............................................................................   700
DRTer07   ...T................T...............................................................................   700
G1Ter08   ...T................T...............................................................................   700
G1Ter12   ...T................T...............................................................................   700
G1Ter02   ...T................T...............................................................................   700
DRTer01   ...A................C...................................A...........................................   700
DRTer05   ...A................C...................................A................................A..........   700
DRTer10   ...A................C...................................A................................A..........   700
DRTer03   ...A................C...................................A................................A..........   700
DRTer08   ...A................C...................................A................................A..........   700
DRTer04   ...A................C...................................A.G..............................A..........   700
DRTer11   ...A................C...................................A................................A..........   700
DRTer06   ...A................C...................................A................................A..........   700
DRTer09   ...A................C...................................A................................A..........   700
DRTer12   ...A................C...................................A................................A..........   700
DRTer13   ...A................C...................................A................................A..........   700
DRTer02   ...T................C...................................A...........................................   700
DRTer14   ...T................C...................................A...........................................   700
G1Ter10   ...T................T...............................................................................   700
```

```
Consensus AGCTTCCATTGCATTGGAGAATGCCAAGGTTAGAGGCAAGGTGGTTCATAGACGTATACGAGAAAAGACAGGACATGAATCCTATCTTGCTGGAGTTTGC   800
G1Ter01   ....................................................................................................   800
G1Ter03   .....................................................A.G............................................   800
G1Ter07   ....................................................................................................   800
G1Ter09   .........................................................G..........................................   800
G1Ter11   ....................................................................................................   800
G1Ter04   .....................................................A.G............................................   800
G1Ter06   .....................................................A.G............................................   800
G1Ter05   .....................................................A.......................T......................   800
DRTer07   .....................................................A.......................T......................   800
G1Ter08   ....................................................................................................   800
G1Ter12   .....................................................A.G...........G................................   800
G1Ter02   .........................T...........................A...........................................C..   800
DRTer01   G...................................................................................................   800
DRTer05   ....................................................................................................   800
DRTer10   ....................................................................................................   800
DRTer03   ....................................................................................................   800
DRTer08   ....................................................................................................   800
DRTer04   ....................................................................................................   800
DRTer11   ....................................................................................................   800
DRTer06   ..................................................................................C.................   800
DRTer09   ....................................................................................................   800
DRTer12   ....................................................................................................   800
DRTer13   ....................................................................................................   800
DRTer02   ............................................................G.......................................   800
DRTer14   ....................................................................--------------------------------   768
G1Ter10   .....................................................A.G............................................   800

Consensus TAAATTGGACTTCAATATGGTACAAGCCACACACCAGGAGGATCTAARACACATGTCTAGCTGGTGGAGGAGTACACGCCTGGGAGAAAAGTTGAAMTTT   900
G1Ter01   ...............................................A................................................A...   900
G1Ter03   ...............................................A................................................A...   900
G1Ter07   ...............................................A................................................A...   900
G1Ter09   ...............................................A................................................A...   900
G1Ter11   ...............................................A................................................A...   900
G1Ter04   ...............................................A................................................A...   900
G1Ter06   ...............................................A................................................A...   900
G1Ter05   ...............................................A................................................A...   900
DRTer07   ...............................................A................................................A...   900
G1Ter08   ...............................................A................................................A...   900
G1Ter12   ...............................................A................................................A...   900
G1Ter02   ...............................................A................................................A...   900
DRTer01   ..........................................C....G................................................A...   900
DRTer05   ...............................................G................................................A...   900
DRTer10   .............G.................................G....G....A...........T..........................C...   900
DRTer03   ...............................................G.........A...........T..........................C...   900
DRTer08   ...............................................G.........A...........T..........................C...   900
DRTer04   ...............................................G.........A...........T..........................C...   900
DRTer11   ...............................................G.........A...........T..........................C...   900
DRTer06   ...............................................G.........A...........T..........................C...   900
DRTer09   ...............................................G.........A...........T..........................C...   900
DRTer12   ...............................................G.........A...........T..........................C...   900
DRTer13   ...............................................G.........A......C....T..........................C...   900
DRTer02   ...............................................G................................................C...   900
DRTer14   ----------------------------------.............G................................................A...   834
G1Ter10   ...............................................A................................................A...   900

Consensus GCTAGGGACAGGCTGATGGAGAATTTCCTATGGACTGTGGGAGTGATATTCGAGCCTCAGTATGGATATTGTAGGAGAATGYTCACGAAGGTTRRCACAY  1000
G1Ter01   .................................................................................C...........GG....T  1000
G1Ter03   .................................................................................CC..........GG....T  1000
G1Ter07   .................................................................................C...........GG....T  1000
G1Ter09   .................................................................................C...........GG....T  1000
G1Ter11   .................................................................................C...........GG....T  1000
G1Ter04   .................................................................................C...........GG....T  1000
G1Ter06   .................................................................................C...........GG....T  1000
G1Ter05   .................................................................................C...........GG....T  1000
DRTer07   .................................................................................C...........GG....T  1000
G1Ter08   .................................................................................C...........GG....T  1000
G1Ter12   .................................................................................C...........GG....T  1000
G1Ter02   .................................................................................C...........GG....T  1000
DRTer01   ...........................................A......T...................A......G...TC..........AA...GC  1000
DRTer05   ..................................................T...................A......G...TC..........AA...GC  1000
DRTer10   ..................................................T...................A......G...TC..........AA...GC  1000
DRTer03   .................................................................................TC...A......AA....C  1000
DRTer08   .................................................................................TC...A......AA....C  1000
DRTer04   .................................................................................TC...A......AA....C  1000
DRTer11   .................................................................................TC...A......AA....C  1000
DRTer06   .................................................................................TC...A......AA....C  1000
DRTer09   .................................................................................TC...A......AA.T..C  1000
DRTer12   .................................................................................TC...A.G....AA....C  1000
DRTer13   .................................................................................TC...A......AA.T..C  1000
DRTer02   .................................................................................TC...A......AA....C  1000
DRTer14   ......................----------------------------T...................A......G...TC..........AA...GC   906
G1Ter10   .................................................................................C......------------   988

Consensus TCATAACRATAATTGATGATGTTTACGACGTTTATGGTACATTGGATGAACTYGAGCTTTTTACAGATGCTGTTGACAGGTGGGATATCAATGCAATGGA  1100
G1Ter01   .......A............................................T...............................................  1100
G1Ter03   .......A.........................................G..T...............................................  1100
G1Ter07   .......A............................................T..........................................G....  1100
G1Ter09   .......A............................................T...............................................  1100
G1Ter11   .......A............................................T...............................................  1100
G1Ter04   .......A............................................T...............................................  1100
G1Ter06   .......A............................................T...............................................  1100
G1Ter05   .......A............................................T...............................................  1100
DRTer07   .......A............................................T...............................................  1100
G1Ter08   .......A............................................T....-..........................................  1099
G1Ter12   .......A............................................T............................-..................  1099
G1Ter02   .......A............................................T...............................................  1100
DRTer01   .......T............................................T................T..............................  1100
DRTer05   .......T............................................T...............................................  1100
DRTer10   .......T............................................T...............................................  1100
DRTer03   .......G.................................A..........C................T..............................  1100
DRTer08   .......G.................................A..........C................T..............................  1100
DRTer04   .......G.................................A..........C..................................A............  1100
DRTer11   .......G.................................A..........C................T..............................  1100
DRTer06   .......G.................................A..........C................T..............................  1100
DRTer09   .......G.................................A..........C................T..............................  1100
DRTer12   .......G.................................A..........C................T..............................  1100
DRTer13   .......G.................................A..........C................T..............................  1100
DRTer02   .......G.................................A..........C................T..............................  1100
DRTer14   .......T............................................T...............................................  1006
G1Ter10   -----------------------------------------------------------------------------.......................  1011
```

```
Consensus TCCACTTCCAGARTACATGAAGTTGTGCTTCCTTGCTCTCTACAACTCCACTAATGAAATGGCTTATGATGCTCTCAAGGAACATGGTTTACASATMATT  1200
G1Ter01   ............A................................................................................G..A...  1200
G1Ter03   ............A................................................................................G..A...  1200
G1Ter07   ............A................................................................................G..A...  1200
G1Ter09   ............A................................................................................G..A...  1200
G1Ter11   ............A................................................................................G..A...  1200
G1Ter04   ............A................................................................................G..A...  1200
G1Ter06   ............A................................................................................G..A...  1200
G1Ter05   ............A.............................................................C..................G..A...  1200
DRTer07   ............A.............................................................C..................G..A...  1200
G1Ter08   ............A................................................................................G..A...  1199
G1Ter12   ............A................................................................................G..A...  1199
G1Ter02   ............A................................................................................C..C...  1200
DRTer01   ............G................................................................................C..CG..  1200
DRTer05   ............T..T..T.................C..........................C...........T.................C..C...  1200
DRTer10   ............T..T..T.................C..........................C...........T.................C..C...  1200
DRTer03   ............G................................................................................C..CG..  1200
DRTer08   ............G......................................C.........................................C..CG..  1200
DRTer04   ............G........T.......................................................................C..CG..  1200
DRTer11   ............G................................................................................C..CG..  1200
DRTer06   ............G................................................................................C..CG..  1200
DRTer09   ............G..........A.....................................................................C..CG..  1200
DRTer12   ............G................................................................................C..CG..  1200
DRTer13   ............G................................................................................C..CG..  1200
DRTer02   ............G................................................................................C..CG..  1200
DRTer14   ............T..T..T.................C..........................C...........T.................C..C...  1106
G1Ter10   ............A................................................................................G..A...  1111

Consensus TCCTATCTTAGAAAAGY-----------------------------------------------------------------------------------  1217
G1Ter01   ................T-----------------------------------------------------------------------------------  1217
G1Ter03   ................T-----------------------------------------------------------------------------------  1217
G1Ter07   ................T-----------------------------------------------------------------------------------  1217
G1Ter09   ................T-----------------------------------------------------------------------------------  1217
G1Ter11   ................T-----------------------------------------------------------------------------------  1217
G1Ter04   ................T-----------------------------------------------------------------------------------  1217
G1Ter06   ................T-----------------------------------------------------------------------------------  1217
G1Ter05   ................T-----------------------------------------------------------------------------------  1217
DRTer07   ................T-----------------------------------------------------------------------------------  1217
G1Ter08   ................T-----------------------------------------------------------------------------------  1216
G1Ter12   ................T-----------------------------------------------------------------------------------  1216
G1Ter02   ................T-----------------------------------------------------------------------------------  1217
DRTer01   ................C-----------------------------------------------------------------------------------  1217
DRTer05   .T..............C-----------------------------------------------------------------------------------  1217
DRTer10   .T..............C-----------------------------------------------------------------------------------  1217
DRTer03   ................C-----------------------------------------------------------------------------------  1217
DRTer08   ................C-----------------------------------------------------------------------------------  1217
DRTer04   ................C-----------------------------------------------------------------------------------  1217
DRTer11   ................C-----------------------------------------------------------------------------------  1217
DRTer06   ................C-----------------------------------------------------------------------------------  1217
DRTer09   ................C-----------------------------------------------------------------------------------  1217
DRTer12   ................C-----------------------------------------------------------------------------------  1217
DRTer13   ................C-----------------------------------------------------------------------------------  1217
DRTer02   ................C-----------------------------------------------------------------------------------  1217
DRTer14   .T..............C-----------------------------------------------------------------------------------  1123
G1Ter10   ................TGGTATTATCTGCAACTCTGAACTCATTCCATTATATGCTGTTTACATGCATGTGGTACTCTTAACTCATGTTATTATGATTAA  1211

Consensus -------GTGGTCAGACTTATGTAAATCTTWCTTACTGGAGGCAAAGTGGTACTACAGTGGATATAMACCAASCCTACAAGAATACATTAGCAATGCATG  1310
G1Ter01   -------.......................T...................................A.....C...........................  1310
G1Ter03   -------.......................T...................................A.....C...........................  1310
G1Ter07   -------.......................T...................................A.....C...........................  1310
G1Ter09   -------.......................T...................................A.....C...........................  1310
G1Ter11   -------.......................T...................................A.....C...........................  1310
G1Ter04   -------.......................T...................................A.....C...........................  1310
G1Ter06   -------......G................T...................................A.....C...........................  1310
G1Ter05   -------.............C.........T...................................A.....C...........................  1310
DRTer07   -------.............C.........T...................................A.....C...........................  1310
G1Ter08   -------.......................T...................................A.....C...........................  1309
G1Ter12   -------.......................T...................................A.....C...........................  1309
G1Ter02   -------.......................T...................................A.....C...........................  1310
DRTer01   -------.....................C.A............................A......C.....G......................T....  1310
DRTer05   -------....G..................A........................T..........C.....G......................T....  1310
DRTer10   -------....G..................A........................T..........C.....G......................T....  1310
DRTer03   -------.....................C.A.......................G....A......C.....G......................T....  1310
DRTer08   -------.....................C.A............................A......C.....G......................T....  1310
DRTer04   -------.....................C.A............................A......C.....G......................T....  1310
DRTer11   -------.....................C.A............................A......C.....G......................T....  1310
DRTer06   -------.....................C.A........................T..........C.....G......................T....  1310
DRTer09   -------.....................C.A............................A......C.....G......................T....  1310
DRTer12   -------.....................C.A............................A......C.....G......................T....  1310
DRTer13   -------.....................C.A............................A......C.....G......................T....  1310
DRTer02   -------.....................C.A........................T..........C.....G........G.............T....  1310
DRTer14   -------.....................C.A............................A......C.....G......................T....  1216
G1Ter10   TCCTTCA.......................T...................................A.....C...........................  1311

Consensus GATTTCRATRTCAGGTCCTGTARTACTAGTCCAYGCYTATTTTYTTGTCRCAAATCCAATCACCAAGGAGGCCTTGCAATCCTTAGAGAGATATCACAAT  1410
G1Ter01   ......G..G............G..........C..T......T.....A..................................................  1410
G1Ter03   ......G..G............G..........C..T......T.....A..................................................  1410
G1Ter07   ......G..G............G..........C..T......T.....A..................................................  1410
G1Ter09   ......G..G............G..........C..T......T.....A..................................................  1410
G1Ter11   ......G..G............G..........C..T......T.....A..................................................  1410
G1Ter04   ......G..G............G..........C..T......T.....A..................................................  1410
G1Ter06   ......G..G............G..........C..T......T.....A..................................................  1410
G1Ter05   ......G..G............G..........C..T......T.....A..................................................  1410
DRTer07   ......G..G............G..........C..T......T.....A..................................................  1410
G1Ter08   ......G..G............G..........C..T......T.....A..................................................  1409
G1Ter12   ......G..G............G..........C..T......T.....A..................................................  1409
G1Ter02   ......G..G............G..........C..T......T.....A..................................................  1410
DRTer01   ......A..A............A..........T..C......C.....G..................................................  1410
DRTer05   ......A..A............A..........T..C......C.....G............................G.....................  1410
DRTer10   ......A..A............A..........T..C......C.....G............................G.....................  1410
DRTer03   ......A..A............A..........T..C......C.....G..................................................  1410
DRTer08   ......A..A............A..........T..C......C.....G..................................................  1410
DRTer04   ......A..A............A..........T..C......C.....G..................................................  1410
DRTer11   ......A..A............A..........T..C......C.....G..................................................  1410
DRTer06   ......A..A......T.....A..........T..C......C.....G..................G.........G.....................  1410
DRTer09   ......A..A............A..........T..C......C.....G..................................................  1410
DRTer12   T.....A..A............A..........T..C......C.....G..................................................  1410
DRTer13   ......A..A............A..........T..C......C.....G..................................................  1410
DRTer02   ......A..A............A..........T..C......C.....G............................G.....................  1410
DRTer14   ......A..A............A..........T..C......C.....G..................................................  1316
G1Ter10   ......G..G............G..........C..T......T.....A..................................................  1411
```

```
Consensus ATTATCCGYTGGTCATCAATGATTTYAAGGCTTTCAGATGATCTGGGAACATCACTG----GATGAGTTGAAAAGAGGAGATGTTCCCAAGTCAATCCAG  1506
G1Ter01   ........T................C...............................----.......................................  1506
G1Ter03   ........C................C...............................----.......................................  1506
G1Ter07   ........C................C...............................----.......................................  1506
G1Ter09   ........T................C...............................----.......................................  1506
G1Ter11   ........T................C...............................----.......................................  1506
G1Ter04   ........C................C...............................----.......................................  1506
G1Ter06   ........C................C...............................----.......................................  1506
G1Ter05   ........C................C...............................----.......................................  1506
DRTer07   ........C................C...............................----.......................................  1506
G1Ter08   ........C................C...............................----.......................................  1505
G1Ter12   ........C................C...............................----.......................................  1505
G1Ter02   ........C................C...............................----.......................................  1506
DRTer01   ........T................T...............................----.......................................  1506
DRTer05   ........T................T...............................----.......................................  1506
DRTer10   ........T................T...............................----.......................................  1506
DRTer03   ........T................T...............................----.......................................  1506
DRTer08   ........T................T...............................----.......................................  1506
DRTer04   ........T................T.............................A.----.......................................  1506
DRTer11   ........T...T............T...............................----.......................................  1506
DRTer06   ........T................T...............................----.......................................  1506
DRTer09   ........T..C.............T...............................----.......................................  1506
DRTer12   ........T.............-..T...............................----.......................................  1505
DRTer13   ........T................T...............................----.......................................  1506
DRTer02   ........T................T...............................----.......................................  1506
DRTer14   ........T................T...............................GTCT.......................................  1416
G1Ter10   ........C................C...............................----.......................................  1507

Consensus TGTTACATGTATGAAACTGGTGCTTCTGAAGAAGACGCCCGTAAACACATAAGCTATTTGATTGRAGRGACATGGAAGAAGCTAAATGAAGATGGAGCTG  1606
G1Ter01   ................................................................A..G.......G........................  1606
G1Ter03   ................................................................A..G................................  1606
G1Ter07   ................................................................A..G................................  1606
G1Ter09   ................................................................A..G................................  1606
G1Ter11   ................................................................A..G................................  1606
G1Ter04   ................................................................A..G................................  1606
G1Ter06   ................................................................A..G................................  1606
G1Ter05   ................................................................A..G.......................G........  1606
DRTer07   ................................................................A..G.......................G........  1606
G1Ter08   ................................................................A..G................................  1605
G1Ter12   ................................................................A..G................................  1605
G1Ter02   ................................................................A..G................................  1606
DRTer01   .................................................C..............G..A................................  1606
DRTer05   .................................................C..............G..A................................  1606
DRTer10   .................................................C..............G..A........................C.......  1606
DRTer03   .................................................C..............G..A................................  1606
DRTer08   .................................................C..............G..A................................  1606
DRTer04   .................................................C..............G..A................................  1606
DRTer11   .................................................C..............G..A................................  1606
DRTer06   .................................................C..............G..A................................  1606
DRTer09   .................................................C..............G..A................................  1606
DRTer12   .................................................C..............G..A................................  1605
DRTer13   ...................................A............................T..AC...............................  1606
DRTer02   ...................................A............................T..AC...............................  1606
DRTer14   ...................................A............................T..AC...............................  1516
G1Ter10   ................................................................A..G................................  1607

Consensus TAGAGTCTCCATTCCCTGAAACWTTTATTAGAATTGCAAYGAATYTTGCTAGGATGGCCCAGTGCATGTATCAGCATGGAGATGGACATGGTATTGAAKA  1706
G1Ter01   ......................T................C...T.....................................................G.  1706
G1Ter03   .............A........T................C...T.....................................................G.  1706
G1Ter07   .............A........T................C...T.........................C...........................G.  1706
G1Ter09   ......................T................C...T.....................................................G.  1706
G1Ter11   ......................T................C...T.....................................................G.  1706
G1Ter04   .............A........T................C...T....................G................................G.  1706
G1Ter06   .............A........T................C...T.....................................................G.  1706
G1Ter05   ......................T................C...T.....................................................G.  1706
DRTer07   ......................T................C...T.....................................................G.  1706
G1Ter08   .............A........T................C...T.....................................................G.  1705
G1Ter12   .............A........T................C...T.....................................................G.  1705
G1Ter02   ......................T................C...T.....................................................G.  1706
DRTer01   ......................A......G.........T...C...........................................C..G......T.  1706
DRTer05   ......................A......G.........T...C...........................................C..G......T.  1706
DRTer10   ......................G......G.........T...C...........................................C..G......T.  1706
DRTer03   ......................A......G.........T...C...........................................C..G......T.  1706
DRTer08   ......................A......G.........T...C...........................................C..G......T.  1706
DRTer04   ......................A......G.........T...C...........................................C..G......T.  1706
DRTer11   ......................G......G.........T...C...........................................C..G......T.  1706
DRTer06   ......................A......G.........T...C...........................................C..G......T.  1706
DRTer09   ......................A......G.........T...C...........................................C..G......T.  1706
DRTer12   ...........-..........A......G.........T...C...........................................C..G......T.  1704
DRTer13   .--...................A......G.........T...C............G.....T..................................T.  1704
DRTer02   .--G..................A......G.........T...C............G.....T..................................T.  1704
DRTer14   .--...................A......G.........T...C............G.....T..................................T.  1614
G1Ter10   ......................T................C...T.....................................................G.  1707

Consensus TGGTGAAACTRAGGATMGAGTGTTGTCACTACTCRTTGAGCCCATTCCCRKMYAKAGNNNASAATAACTAG                                1777
G1Ter01   ..........A.....A.................A..............TTAG.C.AT-G.C...C.....                                1776
G1Ter03   ..........A.....A.................A..............GTAC.T..---.C.....----                                1770
G1Ter07   ..........A.....A.................A..............GTAC.T..---.C.....----                                1770
G1Ter09   ..........A.....A.................A..............GTAC.T..---.C.....----                                1770
G1Ter11   ..........A.....A.................A..............GTAC.T..---.C.....----                                1770
G1Ter04   ..........A.....A.................A..............GTAC.T..---.C.....----                                1770
G1Ter06   ..........A.....A.................A..............GTAC.T..---.C.....----                                1770
G1Ter05   ..........A.....A.................A..............GTAC.T..---.C.....----                                1770
DRTer07   ..........A.....A.................A..............GTAC.T..---.C.....----                                1770
G1Ter08   ..........AG....A.................A..............GTAC.T..---.C.....----                                1769
G1Ter12   ..........A.....A.................A..............TTGG.C.AT-G.C...C.....                                1775
G1Ter02   ..........A.....A.................A..............TTAG.C.AT-G.C...C.....                                1776
DRTer01   .........CG.....C...........T.....G..............AGCTTG..TTCTG.....----                                1773
DRTer05   ..........G.....C...........T.....G..............AGCTTG..TTCTG.....----                                1773
DRTer10   ..........G.....C...........T.....G..............AGCTTG..TTCTG.....----                                1773
DRTer03   ..........G.....C...........T.....G..........C...AGCTTG..TTCTG.....----                                1773
DRTer08   ..........G.....C...........T.....G..............AGCTTG..TTCTG.....----                                1773
DRTer04   ..........G.....C...........T.....G..............AGCTTG..TTCTG.....----                                1773
DRTer11   ..........G.....C...........T....AG........G.....AGCTTG..TTCTG.....----                                1773
DRTer06   ..........G.....C...........T.....G..............AGCTTG..TTCTG.....----                                1773
DRTer09   ..........G.....C...........T.....G..............AGCTTG..TTCTG.....---A                                1774
DRTer12   ..........G.....C...........T.....G..............AGCTTG..TTCTG.....----                                1771
DRTer13   ..........G.....C...........T....AG........G.....AGCTTG..TTCTG.....----                                1771
DRTer02   ..........G.....C...........T.....G..............AGCTTG..TTCTG.....----                                1771
DRTer14   ..........G.....C...........T....AG........G.....AGCTTG..TTCTG.....----                                1681
G1Ter10   ..........A.....A.................A..............GTAC.T..---.C.....----                                1771
```

b

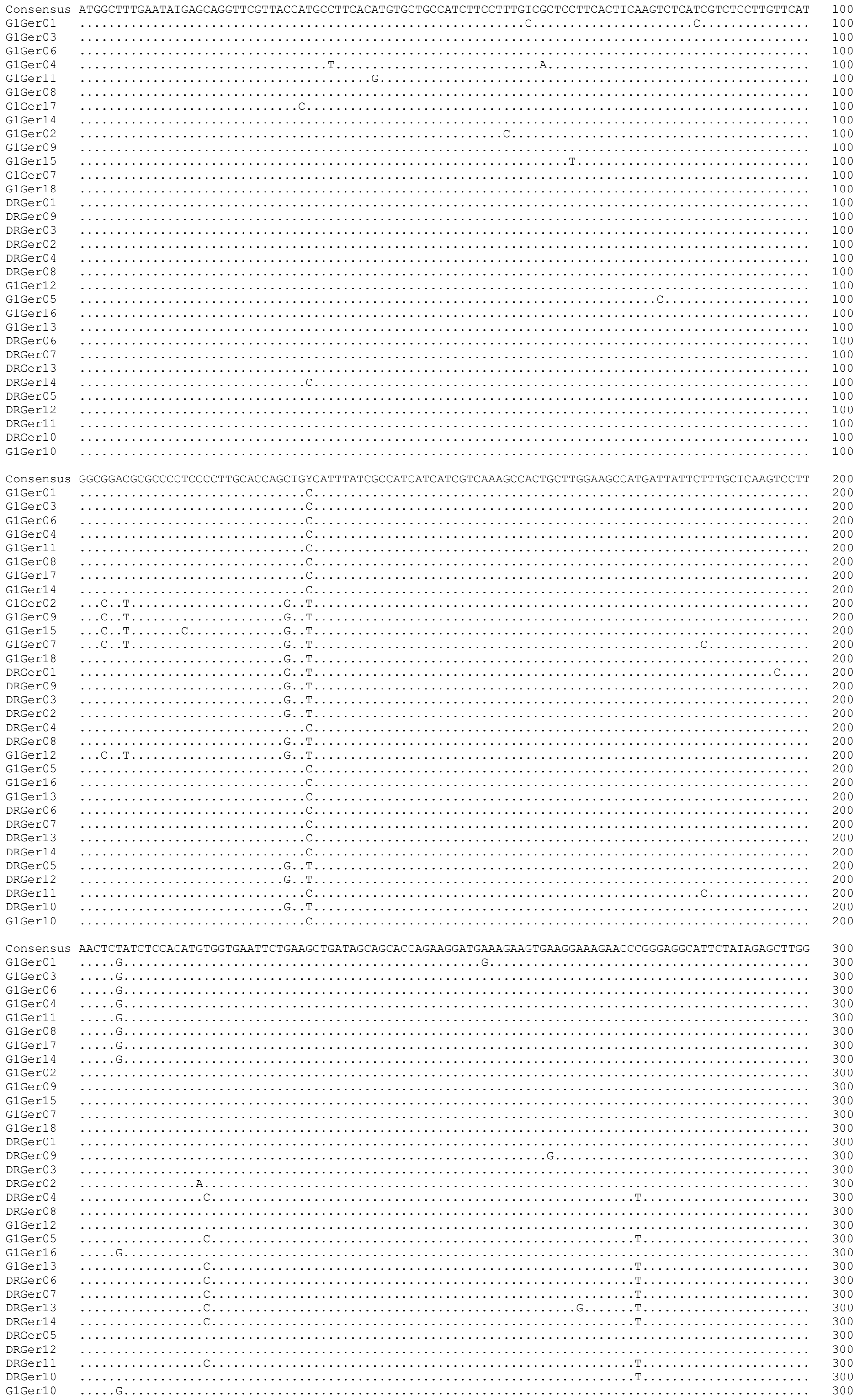
Consensus ATGGCTTTGAATATGAGCAGGTTCGTTACCATGCCTTCACATGTGCTGCCATCTTCCTTTGTCGCTCCTTCACTTCAAGTCTCATCGTCTCCTTGTTCAT 100
Consensus GGCGGACGCGCCCCTCCCCTTGCACCAGCTGYCATTTATCGCCATCATCATCGTCAAAGCCACTGCTTGGAAGCCATGATTATTCTTTGCTCAAGTCCTT 200
Consensus AACTCTATCTCCACATGTGGTGAATTCTGAAGCTGATAGCAGCACCAGAAGGATGAAAGAAGTGAAGGAAAGAACCCGGGAGGCATTCTATAGAGCTTGG 300

```
Consensus GATTCAAGGGCTGCTATGGAGATGGTAGACACAGTTGAGAGGCTAGGTCTATCATACCATTTTGAAGACGAGATAAATGCACTAYTGCAAAGGTTCTGCG   400
G1Ger01   .............................................................................A......C...............   400
G1Ger03   .............................................................................A......C...........C...   400
G1Ger06   .............................................................................A......C...............   400
G1Ger04   .............................................................................A......C...............   400
G1Ger11   .............................................................................A......C...............   400
G1Ger08   .............................................................................A......C...............   400
G1Ger17   .............................................................................A......C...............   400
G1Ger14   .............................................................................A......C...............   400
G1Ger02   ..............................................A...............C....G................T...............   400
G1Ger09   ...................................................................G................T...............   400
G1Ger15   ...................................................................G................T...............   400
G1Ger07   ...................................................................G................T...............   400
G1Ger18   ...................................................................G................T...............   400
DRGer01   ...................................................................G................T...............   400
DRGer09   ...................................................................G................T...............   400
DRGer03   ...................................................................G................T...............   400
DRGer02   ...................................................................G................T...............   400
DRGer04   ....................................................................................T...............   400
DRGer08   ...................................................................G................T...............   400
G1Ger12   .....................G.......................................................A......C...............   400
G1Ger05   ....................................................................................T...............   400
G1Ger16   .....................G.......................................................A......C...............   400
G1Ger13   ....................................................................................T...............   400
DRGer06   ....................................................................................T...............   400
DRGer07   ....................................................................................T...............   400
DRGer13   ....................................................................................T...............   400
DRGer14   ....................................................................................T...............   400
DRGer05   ................................G..................................G................T...............   400
DRGer12   ......G............................................................G................T...............   400
DRGer11   ............................................G.......................................T...............   400
DRGer10   ....................................................................................T...............   400
G1Ger10   .............................................................................A......C...............   400

Consensus ACTGGAATGCCAGTGAAGATCTCTTCACCACTGCCCTTCGTTTCCGGTTGCTTCGCCAAAATGGATTCCCAACTCATTCTGATGTTTTTGGCAAGTTCAT   500
G1Ger01   ........T.......................T...................................................................   500
G1Ger03   ........T.......................T...................................................................   500
G1Ger06   ........T.......................T...................................................................   500
G1Ger04   ........T.......................T...................................................................   500
G1Ger11   ........T.......................T...................................................................   500
G1Ger08   ........T.......................T...................................................................   500
G1Ger17   ........T.......................T...................................................................   500
G1Ger14   ........T.......................T...................................................................   500
G1Ger02   ............................T.......................................................................   500
G1Ger09   ............................T.......................................................................   500
G1Ger15   ............................T.......................................................................   500
G1Ger07   ............................T.......................................................................   500
G1Ger18   ............................T.......................................................................   500
DRGer01   ............................T.......................................................................   500
DRGer09   ............................T.......................................................................   500
DRGer03   ............................T.......................................................................   500
DRGer02   ............................T.......................................................................   500
DRGer04   ....................................................................................................   500
DRGer08   ............................T.......................................................................   500
G1Ger12   ........T.......................T...................................................................   500
G1Ger05   ....................................................................................................   500
G1Ger16   ........T.......................T...................................................................   500
G1Ger13   ....................................................................................................   500
DRGer06   ....................................................................................................   500
DRGer07   ....................................................................................................   500
DRGer13   ....................................................................................................   500
DRGer14   .............C......................................................................................   500
DRGer05   ............................T.......................................................................   500
DRGer12   ............................T.......................................................................   500
DRGer11   ....................................................................................................   500
DRGer10   ....................................................................................................   500
G1Ger10   ........T.................G.....T...................................................................   500

Consensus GGATAAGAATGGAAAATTCAAGGAAAGCTTGACTGAAGACATATGGGGTATGCTGAGTTTACATGAGGCATCTCACTTGGGGGCCAAAAATGAAGAAGTA   600
G1Ger01   ....................................................................................................   600
G1Ger03   ....................................................................................................   600
G1Ger06   ....................................................................................................   600
G1Ger04   ....................................................................................................   600
G1Ger11   ....................................................................................................   600
G1Ger08   ....................................................................................................   600
G1Ger17   ....................................................................................................   600
G1Ger14   ............................................................................C.......................   600
G1Ger02   ....................................................................................................   600
G1Ger09   ....................................................................................................   600
G1Ger15   ....................................................................................................   600
G1Ger07   ....................................................................................................   600
G1Ger18   ....................................................................................................   600
DRGer01   ....................................................................................................   600
DRGer09   ....................................................................................................   600
DRGer03   ....................................................................................................   600
DRGer02   .........................................................................................G..........   600
DRGer04   ....................................................................................................   600
DRGer08   ....................................................................................................   600
G1Ger12   ....................................................................................................   600
G1Ger05   ................C...................................................................................   600
G1Ger16   ....................................................................................................   600
G1Ger13   ................C...................................................................................   600
DRGer06   ....................................................................................................   600
DRGer07   ....................................................................................................   600
DRGer13   ....................................................................................................   600
DRGer14   ....................................................................................................   600
DRGer05   ....................................................................................................   600
DRGer12   ....................................................................................................   600
DRGer11   ....................................................................................................   600
DRGer10   ....................................................................................................   600
G1Ger10   .................C..................................................................................   600
```

```
Consensus TTAGCAGAAGCCAAGGAGTTCACAAGGATTCACCTCATACAGTCTATGCCGCACATGGAACCCCATTTTAGCAGCCATGTCGGCCGGGCACTCGAGCTTC   700
G1Ger01   ..................................................................................................C.   700
G1Ger03   ....................................................................................................   700
G1Ger06   ....................................................................................................   700
G1Ger04   ....................................................................................................   700
G1Ger11   ....................................................................................................   700
G1Ger08   ....................................................................................................   700
G1Ger17   ....................................................................................................   700
G1Ger14   ....................................................................................................   700
G1Ger02   ....................................................................................................   700
G1Ger09   ....................................................................................................   700
G1Ger15   ............................................C.......................................................   700
G1Ger07   .....................................C..............................................................   700
G1Ger18   ....................................................................................................   700
DRGer01   ....................................................................................................   700
DRGer09   ....................................................................................................   700
DRGer03   ....................................................................................................   700
DRGer02   ....................................................................................................   700
DRGer04   ....................................................................................................   700
DRGer08   ....................................................................................................   700
G1Ger12   ....................................................................................................   700
G1Ger05   ......................................G.....C.......................................................   700
G1Ger16   ....................................................................................................   700
G1Ger13   ......................................G.............................................................   700
DRGer06   ....................................................................................................   700
DRGer07   ....................................................................................................   700
DRGer13   ....................................................................................................   700
DRGer14   ....................................................................................................   700
DRGer05   ....................................................................................................   700
DRGer12   ....................................................................................................   700
DRGer11   ..........................................................T.........................................   700
DRGer10   ....................................................................................................   700
G1Ger10   ....................................................................................................   700

Consensus CCAGGCACTTGAGGATGGCGAGGTTGGAGGCTAGAAACTATATAGRCGAATATAGCAGGGAAAGCAACCCGAACTYAGCTCTTCTAGAGCTAGCAAAATT   800
G1Ger01   ..............................T..............G......C......................T........................   800
G1Ger03   ..............................T..............G.............................T........................   800
G1Ger06   ...............C..............T..............G.............................T........................   800
G1Ger04   ..............................T..............G.............................T........................   800
G1Ger11   ..............................T..............G.............................T........................   800
G1Ger08   .............................................A.............................C...............G........   800
G1Ger17   ..............................T..............G.............................T........................   800
G1Ger14   ..............................T.........A....G.............................T........................   800
G1Ger02   .............................................A......C......................C...............G........   800
G1Ger09   .............................C...............A.............................C...............G........   800
G1Ger15   .............................................A.............................C...............G........   800
G1Ger07   .............................................A....C........................C...............G........   800
G1Ger18   .............................................A.............................C...............G........   800
DRGer01   .............................................A.............................C...............G........   800
DRGer09   .............................................A.............................C...............G........   800
DRGer03   .............................................A.............................C...............G........   800
DRGer02   .............................................A.............................C...............G........   800
DRGer04   .............................................A.............................C...............G........   800
DRGer08   .............................................A.............................C...............G........   800
G1Ger12   .............................................A.............................C...............G........   800
G1Ger05   ..................T..........................G.............................T....T...................   800
G1Ger16   ..............................T..............G.............................T........................   800
G1Ger13   ..................T..........................G.............................T....T...................   800
DRGer06   ..................T..........................G.............................T....T...................   800
DRGer07   ..................T..........................G.............................T....T...................   800
DRGer13   ..................T..........................G.............................T....T...................   800
DRGer14   ..................T..........................G.............................T....T...................   800
DRGer05   .............................................A.............................C...............G........   800
DRGer12   .............................................A.............................C...............G........   800
DRGer11   ..................T..........................G.............................T....T...................   800
DRGer10   .............................................A.............................C...............G........   800
G1Ger10   ..............................T..............G.............................T........................   800

Consensus GGATTTCGACATGGTTCAATCACTGCACCAGAAAGAGTTGGCTGAGATAGT-------------------------AAGGTGGTGGAAACAGTTGGGTCT   875
G1Ger01   ...........................................A.......-------------------------........................   875
G1Ger03   ...................................................-------------------------........................   875
G1Ger06   ...................................................-------------------------........................   875
G1Ger04   ...................................................-------------------------........................   875
G1Ger11   ...................................................-------------------------........................   875
G1Ger08   ...................................................-------------------------........................   875
G1Ger17   ...................................................-------------------------........................   875
G1Ger14   ...................................................-------------------------........................   875
G1Ger02   ...................................................-------------------------........................   875
G1Ger09   ...................................................-------------------------........................   875
G1Ger15   ...................................................-------------------------........................   875
G1Ger07   ..........................G........................-------------------------........................   875
G1Ger18   ...................................................-------------------------..C.....................   875
DRGer01   ...................................................-------------------------........................   875
DRGer09   ...................................................-------------------------........................   875
DRGer03   ...................................................-------------------------..............G.........   875
DRGer02   ...................................................-------------------------........................   875
DRGer04   ...................................................-------------------------........................   875
DRGer08   ...................................................-------------------------........................   875
G1Ger12   ...................................................-------------------------........................   875
G1Ger05   ...................................................-------------------------........................   875
G1Ger16   ...................................................AAGGCCATCCATGTTCAATTCTATA........................   900
G1Ger13   ...................................................-------------------------........................   875
DRGer06   .................................................T.-------------------------........................   875
DRGer07   .................................................T.-------------------------........................   875
DRGer13   .................................................T.-------------------------........................   875
DRGer14   .................................................T.-------------------------........................   875
DRGer05   .................G...............................T.-------------------------........................   875
DRGer12   ...................................................-------------------------........................   875
DRGer11   .................................................T.-------------------------........................   875
DRGer10   .........................................T.........-------------------------........................   875
G1Ger10   ...................................................-------------------------........................   875
```

```
Consensus TGTTGATAAGCTTGATTTTGCTCGAGACCGGCCGATGGAGTGCTTCTTATGGACAGTGGGGATATTTCCAGATCCCAGGCATTCAAGCTGCCGCATCGAA   975
G1Ger01   ....................................................................................................   975
G1Ger03   ....................................................................................................   975
G1Ger06   ....................................................................................................   975
G1Ger04   .....................................................................................G..............   975
G1Ger11   ....................................................................................................   975
G1Ger08   ....................................................................................................   975
G1Ger17   ....................................................................................................   975
G1Ger14   .........................................................A..........................................   975
G1Ger02   ....................................................................................................   975
G1Ger09   ....................................................................................................   975
G1Ger15   ....................................................................................................   975
G1Ger07   ....................................................................................................   975
G1Ger18   ....................................................................................................   975
DRGer01   ....................................................................................................   975
DRGer09   ....................................................................................................   975
DRGer03   ....................................................................................................   975
DRGer02   ....................................................................................................   975
DRGer04   ....................................................................................................   975
DRGer08   ....................................................................................................   975
G1Ger12   ....................................................................................................   975
G1Ger05   ....................................................................................................   975
G1Ger16   ....................................................................................................  1000
G1Ger13   ....................................................................................................   975
DRGer06   ..............................................................................A.....................   975
DRGer07   ..............................................................................A.....................   975
DRGer13   ..............................................................................A.....................   975
DRGer14   ..............................................................................A.....................   975
DRGer05   ..............................................................................A.....................   975
DRGer12   ....................................................................................................   975
DRGer11   ..............................................................................A.....................   975
DRGer10   ....................................................................................................   975
G1Ger10   ............C.......................................................................................   975

Consensus TTAACCAAGGCCATTGCTATCTTACTAGTTATTGATGATATTTACGACTCTTATGGATCTTTGGACGAGCTTGCTCTCTTCACTGATGCAGTAAAAAGAT  1075
G1Ger01   ....................................................................................................  1075
G1Ger03   ....................................................................................................  1075
G1Ger06   ....................................................................................................  1075
G1Ger04   ....................................................................................................  1075
G1Ger11   ....................................................................................................  1075
G1Ger08   ....................................................................................................  1075
G1Ger17   ......................................................................A.............................  1075
G1Ger14   ....................................................................................................  1075
G1Ger02   ....................................................................................................  1075
G1Ger09   ....................................................................................................  1075
G1Ger15   ....................................................................................................  1075
G1Ger07   ....................................................................................................  1075
G1Ger18   ..............................T......T.G............................................................  1075
DRGer01   ..............................T........G...............................................A............  1075
DRGer09   ..............................T........G............................................................  1075
DRGer03   ..............................T........G............................................................  1075
DRGer02   ..............................T........G............................................................  1075
DRGer04   ..............................T........G.......................................................G....  1075
DRGer08   ..............................T........G............................................................  1075
G1Ger12   ....................................................................................................  1075
G1Ger05   ....................................................................................................  1075
G1Ger16   ....................................................................................................  1100
G1Ger13   ....................................................................................................  1075
DRGer06   ....................................................................................................  1075
DRGer07   ....................................................................................................  1075
DRGer13   ....................................................................................................  1075
DRGer14   ....................................................................................................  1075
DRGer05   ....................................................................................................  1075
DRGer12   ..............................T........G............................................................  1075
DRGer11   ....................................................................................................  1075
DRGer10   ..............................T........G............................................................  1075
G1Ger10   ....................................................................................................  1075

Consensus GGGATCTTGGTGCAATGGATCAGCTCCCTGAGTACATGAAGATATGTTACATGGCATTGTACAACACTACTAATGACATTGCATACAGGATCTTGAAGGA  1175
G1Ger01   ....................................................................................................  1175
G1Ger03   ....................................................................................................  1175
G1Ger06   ....................................................................................................  1175
G1Ger04   ....................................................................................................  1175
G1Ger11   .........................T..........................................................................  1175
G1Ger08   ...........................................G........................................................  1175
G1Ger17   ....................................................................................................  1175
G1Ger14   ....................................................................................................  1175
G1Ger02   ....................................................................................................  1175
G1Ger09   ....................................................................................................  1175
G1Ger15   ....................................................................................................  1175
G1Ger07   ....................................................................................................  1175
G1Ger18   ....................................................................................................  1175
DRGer01   ....................................................................................................  1175
DRGer09   ....................................................................................................  1175
DRGer03   ........................................................C...........................................  1175
DRGer02   ....................................................................................................  1175
DRGer04   ....................................................................................................  1175
DRGer08   ....................................................................................................  1175
G1Ger12   ....................................................................................................  1175
G1Ger05   ....................................................................................................  1175
G1Ger16   ....................................................................................................  1200
G1Ger13   ....................................................................................................  1175
DRGer06   ....................................................................................................  1175
DRGer07   .....................................................................................T..............  1175
DRGer13   ....................................................................................................  1175
DRGer14   ....................................................................................................  1175
DRGer05   .........................................................................................G..........  1175
DRGer12   ....................................................................................................  1175
DRGer11   .............G......................................................................................  1175
DRGer10   ....................................................................................................  1175
G1Ger10   ....................................................................................................  1175
```

```
Consensus GCATGGATGGAGCGTCATAGAAGACCTAAAACGAACGTGGATGGACATCTTTGGAGCTTTCCTAGCTGAAGCCCATTGTTTCAAAGGTGGCCATGTACCC  1275
G1Ger01   ....................................................................................................  1275
G1Ger03   ....................................................................................................  1275
G1Ger06   ....................................................................................................  1275
G1Ger04   ....................................................................................................  1275
G1Ger11   ....................................................................................................  1275
G1Ger08   ....................................................................................................  1275
G1Ger17   ....................................................................................................  1275
G1Ger14   .......................................................A............................................  1275
G1Ger02   ....................................................................................................  1275
G1Ger09   ....................................................................................................  1275
G1Ger15   ....................................................................................................  1275
G1Ger07   ....................................................................................................  1275
G1Ger18   ....................................................................................................  1275
DRGer01   ....................................................................................................  1275
DRGer09   ....................................................................................................  1275
DRGer03   ....................................................................................................  1275
DRGer02   ....................................................................................................  1275
DRGer04   ....................................................................................................  1275
DRGer08   ................................-----...............................................................  1270
G1Ger12   ....................................................................................................  1275
G1Ger05   .........................................................................T...................C......  1275
G1Ger16   ......................................................T.............................................  1300
G1Ger13   .........................................................................T...................C......  1275
DRGer06   ......................C..................................................T...................C......  1275
DRGer07   ......................C..................................................T...................C......  1275
DRGer13   ......................C..................................................T...................C......  1275
DRGer14   ......................C..................................................T...................C......  1275
DRGer05   ......................C..................................................T...................C......  1275
DRGer12   .........................................................................T..................GC......  1275
DRGer11   ......................C..................................................T...................C......  1275
DRGer10   ....................................................................................................  1275
G1Ger10   ..................................................................----------------------------------  1241

Consensus TCACTAGAAGAGTATCTAAATAATGCAGTTACCACTGGAGGCACATACATGGCCTTGGTGCATGCCTTTTTTCTTATGGGGCAAGGTGTTACTAGGGAAA  1375
G1Ger01   ............C.......................................................................................  1375
G1Ger03   ............C.......................................................................................  1375
G1Ger06   ............C.............................................................................G.........  1375
G1Ger04   ............C.......................................................................................  1375
G1Ger11   ............CG......................................................................................  1375
G1Ger08   ....................................................................................................  1375
G1Ger17   ....................................................................................................  1375
G1Ger14   ....................................................................................................  1375
G1Ger02   ....................................................................................................  1375
G1Ger09   ....................................................................................................  1375
G1Ger15   ....................................................................................................  1375
G1Ger07   .........................T..........................................................................  1375
G1Ger18   ....................................................................................................  1375
DRGer01   ....................................................................................................  1375
DRGer09   ....................................................................................................  1375
DRGer03   ....................................................................................................  1375
DRGer02   ....................................................................................................  1375
DRGer04   ....................................................................................................  1375
DRGer08   ....................................................................................................  1370
G1Ger12   ....................................................................................................  1375
G1Ger05   ....................................................................................................  1375
G1Ger16   ............C.......................................................................................  1400
G1Ger13   ...................C.............................................T..................................  1375
DRGer06   ...................C.............................................T..................................  1375
DRGer07   ...................C.............................................T..................................  1375
DRGer13   ...................C.............................................T..................................  1375
DRGer14   ...................C.............................................T..................................  1375
DRGer05   ...................C.............................................T..................................  1375
DRGer12   ...................C.............................................T..................................  1375
DRGer11   .....G..G..........C.............................................-..................................  1374
DRGer10   ....................................................................................................  1375
G1Ger10   ----------------------------------------------------------------------------------------------------  1241

Consensus ATATGGCCATGTTGAAGCCCTATCCCAACATCTTCTCCTGTTCAGGGAAAATTCTTCGGCTATGGGATGACTTGGGAACTGCAAGGGAGGAGCAAGAACG  1475
G1Ger01   ...........................G........................................................................  1475
G1Ger03   ....................................................................................................  1475
G1Ger06   ....................................................................................................  1475
G1Ger04   ....................................................................................................  1475
G1Ger11   ....................................................................................................  1475
G1Ger08   ....................................................................................................  1475
G1Ger17   .................................................................T..................................  1475
G1Ger14   .................................................................T..................................  1475
G1Ger02   .................................................................T.....C...............G............  1475
G1Ger09   .................................................................T..................................  1475
G1Ger15   .................................................................T..................................  1475
G1Ger07   .................................................................T..................................  1475
G1Ger18   ...............G....................................................................................  1475
DRGer01   ....................................................................................................  1475
DRGer09   ....................................................................................................  1475
DRGer03   ....................................................................................................  1475
DRGer02   ................................................................................................G...  1475
DRGer04   ....................................................................................................  1475
DRGer08   ..........................G.........................................................................  1470
G1Ger12   .................................................................T................G.................  1475
G1Ger05   ..........................G......................................T..................................  1475
G1Ger16   ....................................................................................................  1500
G1Ger13   ........................................C....................T.......................A...........GA.  1475
DRGer06   ........................................C....................T.......................A...........GA.  1475
DRGer07   ........................................C....................T.......................A...........GA.  1475
DRGer13   ........................................C....................T.......................A...........GA.  1475
DRGer14   ........................................C....................T.......................A...........GA.  1475
DRGer05   ........................................C....................T.......................A...........GA.  1475
DRGer12   ........................................C....................T.......................A...........GA.  1475
DRGer11   ........................................C....................T.......................A...........GA.  1474
DRGer10   ....................................................................................................  1475
G1Ger10   ------------........................................................................................  1329
```

```
Consensus AGGAGATAACGCGTCGAGTATAGAATGCTACAAGCGAGAGCGAGAGCGAGAGATGGATACTGTGTTGGATGATGAAGCCTGCAGGAAACACATAAGGCAG  1575
G1Ger01   ....................................................................................................  1575
G1Ger03   ....................................................................................................  1575
G1Ger06   ....................................................................................................  1575
G1Ger04   ....................................................................................................  1575
G1Ger11   ....................................................................................................  1575
G1Ger08   ....................................................................................................  1575
G1Ger17   ....................................................................................................  1575
G1Ger14   .........................................................................................G..........  1575
G1Ger02   ....................................................................................................  1575
G1Ger09   ................................G...................................................................  1575
G1Ger15   ....................................................................................................  1575
G1Ger07   .....................................................................A..............................  1575
G1Ger18   ....................................................................................................  1575
DRGer01   ....................................................................................................  1575
DRGer09   ....................................................................................................  1575
DRGer03   ....................................................................................................  1575
DRGer02   ....................................................................................................  1575
DRGer04   ....................................................................................................  1575
DRGer08   ....................................................................................................  1570
G1Ger12   ....................................................................................................  1575
G1Ger05   ....................................................................................................  1575
G1Ger16   ....................................................................................................  1600
G1Ger13   ............A...................------...............................A..............................  1569
DRGer06   ................................------...............................A..............................  1569
DRGer07   ................................------...............................A..............................  1569
DRGer13   ................................------...............................A..............................  1569
DRGer14   ................................------...............................A..............................  1569
DRGer05   ................................------...............................A..............................  1569
DRGer12   ................................------...............................A..............................  1569
DRGer11   ................................------........................G......A..............................  1568
DRGer10   ..........................................----------------------------------------------------------  1517
G1Ger10   ....................................................................................................  1429

Consensus ATGATCCAGAGCTTGTGGGTAGAGCTCAATGGGGAGCTGGTTGCTTCGTCTGCATTGCCTCTTTCAATCATCAAAGCTGCCTTTAACCTCTCAAGAACTG  1675
G1Ger01   .............................G......................................................................  1675
G1Ger03   ....................................................................................................  1675
G1Ger06   ....................................................................................................  1675
G1Ger04   ....................................................................................................  1675
G1Ger11   ....................................................................................................  1675
G1Ger08   ....................................................................................................  1675
G1Ger17   ....................................................................................................  1675
G1Ger14   ....................................................................................................  1675
G1Ger02   ....................................................................................................  1675
G1Ger09   ....................................................................................................  1675
G1Ger15   .C..................................................................................................  1675
G1Ger07   ....................................................................................................  1675
G1Ger18   ....................................................................................................  1675
DRGer01   ....................................................................................................  1675
DRGer09   ....................................................................................................  1675
DRGer03   ....................................................................................................  1675
DRGer02   ....................................................................................................  1675
DRGer04   ....................................................................................................  1675
DRGer08   ...................................................................C................................  1670
G1Ger12   .C.........................................T........................................................  1675
G1Ger05   ....................................................................................................  1675
G1Ger16   ....................................................................................................  1700
G1Ger13   ....................................................................................................  1669
DRGer06   ....................................................................................................  1669
DRGer07   ....................................................................................................  1669
DRGer13   ....................................................................................................  1669
DRGer14   ....................................................................................................  1669
DRGer05   ....................................................................................................  1669
DRGer12   ....................................................................................................  1669
DRGer11   ....................................................................................................  1668
DRGer10   --------------------................................................................................  1597
G1Ger10   ....................................................................................................  1529

Consensus CACAAGTCATTTACCAGCATGGAGATGATAACAAGACTTCTAGTGTTGAAGATCATGTACAAGCATTGCTCTTTAGGCCTGTTTCCTCTAATGGGCATGC  1775
G1Ger01   ...........................................C...........................C.......................A....  1775
G1Ger03   ....................................................................................................  1775
G1Ger06   ....................................................................................................  1775
G1Ger04   ....................................................................................................  1775
G1Ger11   ....................................................................................................  1775
G1Ger08   ....................................................................................................  1775
G1Ger17   ....................................................................................................  1775
G1Ger14   ....................................................................................................  1775
G1Ger02   ....................................................................................................  1775
G1Ger09   ....................................................................................................  1775
G1Ger15   ...............G....................................................................................  1775
G1Ger07   ....................................................................................................  1775
G1Ger18   .G..................................................................................................  1775
DRGer01   ....................................................................................................  1775
DRGer09   ....................................................................................................  1775
DRGer03   ....................................................................................................  1775
DRGer02   ....G...............................................................................................  1775
DRGer04   ....................................................................................................  1775
DRGer08   ....................................................................................................  1770
G1Ger12   ....................................................................................................  1775
G1Ger05   ...............................................................................................A....  1775
G1Ger16   ....................................................C...............................................  1800
G1Ger13   .........................................................................................G..........  1769
DRGer06   ....................................................................................................  1769
DRGer07   ....................................................................................................  1769
DRGer13   ....................................................................................................  1769
DRGer14   ....................................................................................................  1769
DRGer05   ....................................................................................................  1769
DRGer12   ....................................................................................................  1769
DRGer11   ....................................................................................................  1768
DRGer10   ....................................................................................................  1697
G1Ger10   ....................................................................................................  1629
```

```
Consensus TCAAATTACCATGCATTAG     1794
G1Ger01   ...................     1794
G1Ger03   ...................     1794
G1Ger06   ...................     1794
G1Ger04   ...................     1794
G1Ger11   ...................     1794
G1Ger08   ............A......     1794
G1Ger17   ...................     1794
G1Ger14   ...................     1794
G1Ger02   ...................     1794
G1Ger09   ...................     1794
G1Ger15   ...................     1794
G1Ger07   ...................     1794
G1Ger18   ...................     1794
DRGer01   ...................     1794
DRGer09   ...................     1794
DRGer03   ...................     1794
DRGer02   ...................     1794
DRGer04   ...................     1794
DRGer08   ...................     1789
G1Ger12   ...................     1794
G1Ger05   ...................     1794
G1Ger16   ...................     1819
G1Ger13   .........A.........     1788
DRGer06   ...................     1788
DRGer07   ...................     1788
DRGer13   ...................     1788
DRGer14   ...................     1788
DRGer05   ...................     1788
DRGer12   ...................     1788
DRGer11   ...................     1787
DRGer10   ...................     1716
G1Ger10   ...................     1648
```

Supplementary Figure 6. Alignments of isolated (**a**) *VvTER* and (**b**) *VvGER* gene copies from *Vitis vinifera* cDNA. Genes isolated from 'Deckrot' are given the suffix DR, while genes isolated from G1-7720 are given the suffix G1. A consensus sequences is given, with dots indicating identity and letters illustrating divergent sequences.

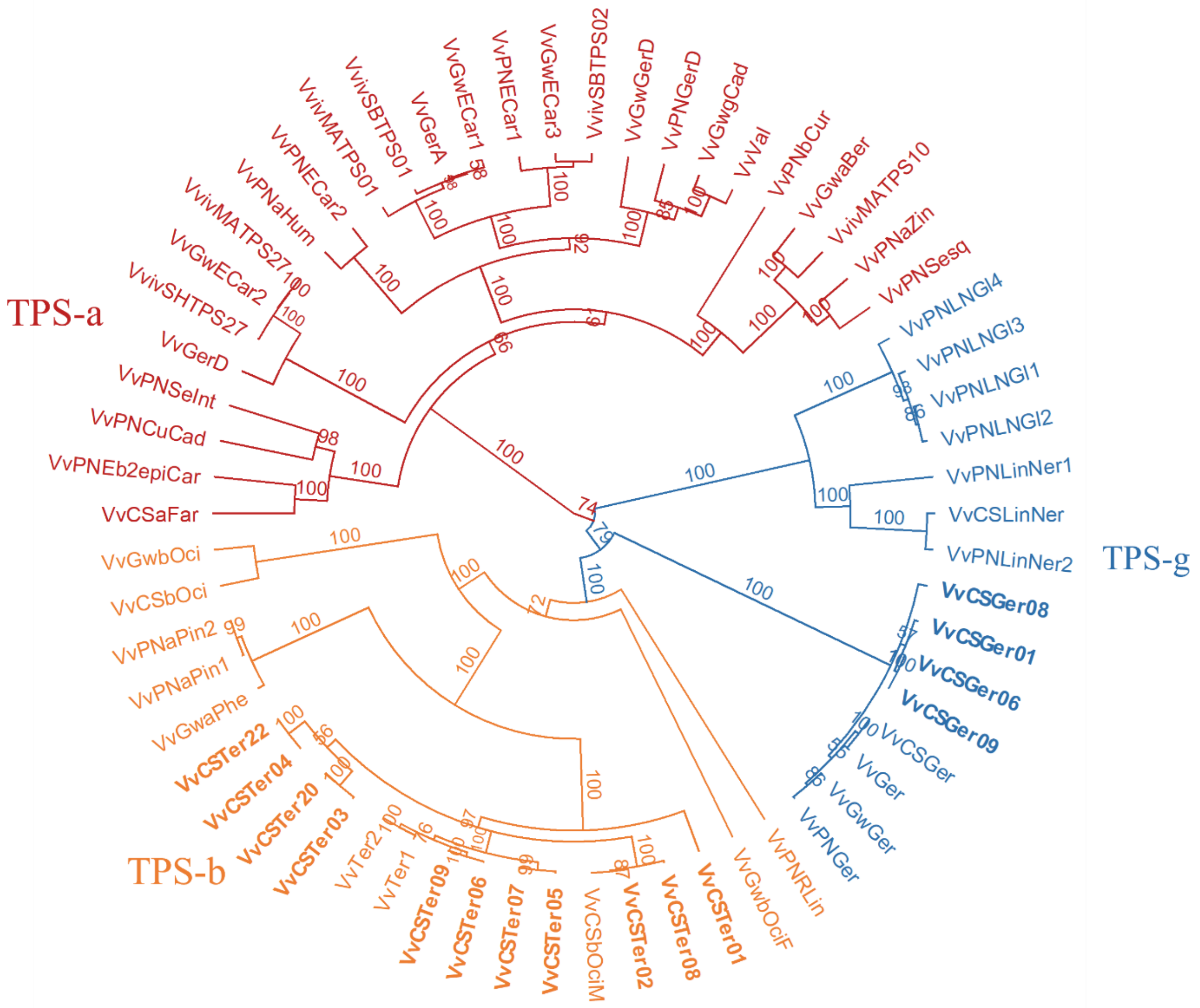

Supplementary Figure 7. Molecular phylogenetic tree for protein sequences of functionally characterised V. vinifera terpene synthases and putative Cabernet Sauvignon VvTer and VvGer genes. Nodes are coloured according to which TPS subfamily the proteins fall under. Putative Cabernet Sauvignon genes are in bold typeface. VvCSTer enzymes are closely related to functionally characterised α-terpineol synthases (VvTer1 and VvTer2), while VvCSGer enzymes are closely related to functionally characterised geraniol synthases (VvGer, VvPNGer and VvGwGer).
